# Sustainable Synthesis of Novel Hydroxylated Tranilast Analogues and Their Bioactivities

**DOI:** 10.3390/molecules31081340

**Published:** 2026-04-19

**Authors:** Angela Maione, Marianna Imparato, Luigi Cirillo, Marco Guida, Emilia Galdiero, Armando Zarrelli, Luigi Longobardo

**Affiliations:** 1 Department of Biology, University of Naples Federico II, 80126 Naples, Italy; angela.maione@unina.it (A.M.); marianna.imparato@unina.it (M.I.); marco.guida@unina.it (M.G.); egaldier@unina.it (E.G.); 2 Division of Urology, Asl Napoli3 Sud, ‘San Leonardo’ Hospital, 80053 Castellammare di Stabia, Italy; cirilloluigi22@gmail.com; 3 Department of Chemical Science, University of Napoli Federico II, Via Cinthia 4, 80126 Napoli, Italy; luilongo@unina.it

**Keywords:** cinnamides, structure–activity relationship, anti-inflammatory, antimicrobial, antiproliferative activity

## Abstract

Tranilast, an anti-allergic drug with well-established anti-inflammatory, antifibrotic, and antiproliferative properties, suffers from poor water solubility and low bioavailability, which limit its therapeutic potential. To improve its pharmacological profile, we designed and synthesized a novel series of hydroxylated Tranilast analogues. The compounds were obtained through a green, single-step coupling reaction between activated methoxy-substituted hydroxycinnamic acids and anthranilic or hydroxyanthranilic acids, using a triethylamine–isobutyl chloroformate system in environmentally friendly solvents. Fifteen derivatives were isolated in good to excellent yields (63–94%) without chromatographic purification. The synthesized compounds were evaluated for antimicrobial, antioxidant, anti-inflammatory, and antiproliferative activities. Several analogues displayed notable antimicrobial effects against *Candida albicans*, *Staphylococcus aureus*, and *Klebsiella pneumoniae*, with minimum inhibitory concentrations as low as 75 µg/mL. Hydroxylated derivatives showed enhanced radical-scavenging activity in DPPH and ABTS assays compared with Tranilast. Selected compounds also demonstrated suggestive antiproliferative effects against LNCaP prostate cancer cells while maintaining low cytotoxicity toward HaCaT keratinocytes, indicating favourable selectivity. Furthermore, some derivatives significantly reduced nitric oxide production in LPS-stimulated HaCaT cells, confirming their anti-inflammatory potential. Overall, hydroxylation proves to be an effective strategy for improving the biological profile of Tranilast, yielding promising candidates for further pharmacological development.

## 1. Introduction

Tranilast, chemically known as *N*-(3,4-dimethoxycinnamoyl)anthranilic acid, was developed as an anti-allergic drug and later gained interest for its diverse pharmacological activities [1,2]. Approved in Japan and South Korea for bronchial asthma and atopic dermatitis, it was subsequently shown to exert anti-fibrotic, anti-proliferative, and anti-inflammatory effects [3,4,5], mainly through modulation of pathways involving TGF-β and PDGF [6]. These properties prompted investigations in clinical trials for keloids, hypertrophic scars, cancer, cardiovascular disorders, and restenosis [7,8,9,10]. However, its clinical use is limited by poor water solubility, low oral bioavailability, and rapid metabolism, which require high and frequent dosing and may lead to adverse effects such as hepatotoxicity and gastrointestinal discomfort [11]. For these reasons, there is considerable interest in developing Tranilast analogues with improved drug-like properties [12]. Introducing hydroxyl groups (–OH) into the parent scaffold is a common medicinal chemistry strategy: in Tranilast analogues, hydroxyl substituents—typically on the anthranilate ring—can improve aqueous solubility, support radical scavenging, and enhance interactions with biological targets through hydrogen bonds [13]. Our goal is to overcome Tranilast’s pharmacokinetic limitations by identifying next-generation derivatives with increased potency and potential applications in cancer, neurodegeneration, and inflammatory conditions.

Tranilast synthesis is straightforward and amenable to analogue preparation. The molecule consists of a cinnamoyl moiety conjugated to an anthranilic acid unit. The most common industrial method involves a one-step condensation between 2-aminobenzoic acid and 3,4-dimethoxycinnamic acid, typically via activation to the corresponding acid chloride, followed by amide bond formation in the presence of a base. Although efficient, this method uses toxic reagents (e.g., SOCl_2_, dichloromethane) and, with phenolic –OH groups, may produce undesired by-products [14]. Alternative multi-step routes for halogenated analogues have been reported, though they often require toxic reagents and give low overall yields [15,16]. While fluorinated derivatives have shown improved in vitro activity, their environmental persistence raises sustainability concerns.

**Figure 1:**
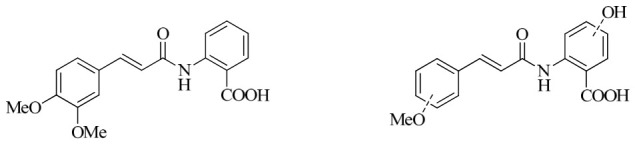
Tranilast and hydroxylated and/or methoxylated analogues.

In this work, we applied an established mixed-anhydride methodology under environmentally friendly conditions to prepare a new series of hydroxylated Tranilast analogues. By varying the methoxy pattern on the cinnamic ring and introducing a hydroxyl group on the anthranilate portion (Figure 1), we obtained the target amides in a single step using environmentally friendly solvents and without laborious purification. Their antioxidant activity, anti-inflammatory effects in LPS-stimulated HaCaT cells, antifungal activity against *Staphylococcus aureus*, *Candida albicans*, and *Klebsiella pneumoniae*, and antiproliferative effects in LNCaP prostate cancer cells were subsequently evaluated.

## 2. Results and Discussion

### 2.1. Chemical Synthesis

**Figure 2:**
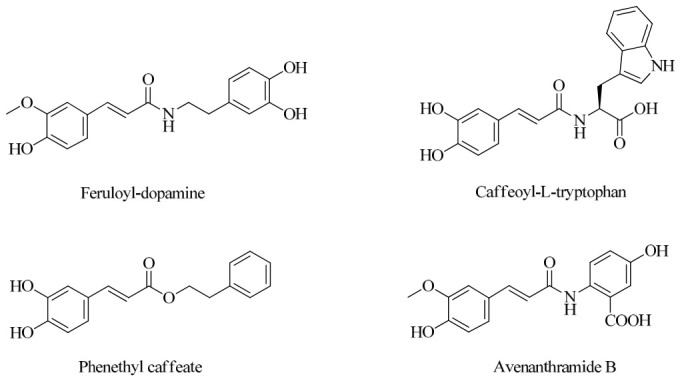
Some natural products derived from hydroxycinnamic acid were prepared using a green synthetic platform.

**Figure 3:**
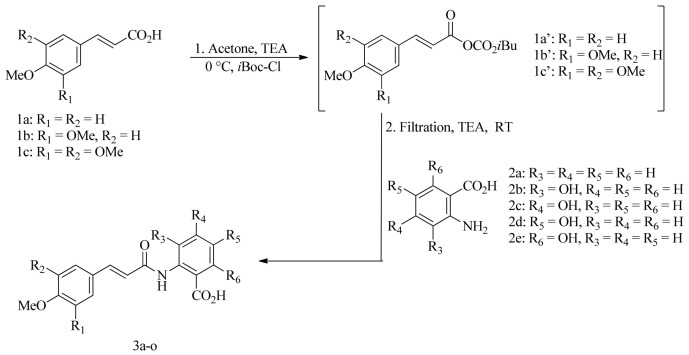
Preparation of hydroxylated Tranilast analogues **3a**–**o** from methoxycinnamic acids **1a**–**c** and anthranilic acid **2a** and its hydroxy-substituted derivatives **2b**–**e**.

The activation of common hydroxycinnamic acids (HCAs) with the established triethylamine–isobutyl chloroformate reagent system in environmentally friendly solvents (water, ethyl acetate, or acetone) has been widely employed to prepare various HCA derivatives, including amides, esters, and alcohols, several of which occur in natural products [17,18] (Figure 2). The coupling of activated HCAs with aromatic amino acids has further allowed the eco-friendly synthesis of natural avenanthramides and numerous related analogues [19]. In this work, a series of hydroxylated Tranilast analogues—classified as *N*-methoxycinnamoyl-hydroxyanthranilic acids—was efficiently prepared in acetone using the triethylamine–isobutyl chloroformate activation system. Specifically, various commercially available methoxycinnamic acids **1a**–**c** were coupled with anthranilic acid **2a** and the four possible monohydroxyanthranilic acids **2b**–**e** (Figure 3), rapidly affording the desired conjugated products under mild and sustainable reaction conditions.

The methoxycinnamic acids **1a**–**c** were activated with the reagent system TEA/*i*Boc-Cl in acetone at 0 °C for 15 min. The resulting suspension, which contained the white precipitate of triethylammonium chloride and the mixed carbonic anhydride of methoxycinnamic acids **1a′**–**c′**, was then filtered, and the filtrate was directly added to the solid aromatic amino acids **2a**–**e**, together with an additional equivalent of triethylamine, as illustrated in Figure 3.

After stirring the reaction mixture at room temperature for 4 h, the solvent was evaporated under reduced pressure. The resulting residue was treated with anhydrous diethyl ether and stirred for 1 h to ensure complete precipitation of the amide products, which contain three polar functional groups. Under these conditions, unreacted starting materials remain in solution, while the desired amides selectively precipitate. The solid was collected by filtration, washed with petroleum ether, and dried under a stream of N_2_(g). Using this procedure, the hydroxylated Tranilast analogues **3a**–**o** (Table 1) were obtained in yields ranging from 63% to 94%.

In most cases, the crude solids were obtained in pure form, as confirmed by TLC and ^1^H-NMR analyses. When minor impurities were present—particularly for compounds **3g**–**i** and **3m**–**o**—further purification was achieved by recrystallization from ethyl acetate/n-hexane mixtures. We observed that anthranilic acid **2a** and hydroxyanthranilic acids **2c** and **2e** provided lower yields than their isomeric counterparts **2b** and **2d**. This can be attributed to the presence of electron-donating substituents in the ortho and para positions relative to the amino group in acids **2b** and **2d**, which increase nucleophilicity through resonance effects, thereby favoring amide bond formation.

These hydroxylated Tranilast analogues show a notable structural resemblance to avenanthramides; in particular, compound **3l** corresponds to a naturally occurring avenanthramide previously isolated and characterized [20].

Compound **3l** was recently synthesized in two steps with a 23% overall yield [16] through *N*-amidation of 5-hydroxyanthranilic acid with Meldrum’s acid in toluene under reflux for several hours. This was followed by the Knoevenagel–Doebner condensation of the resulting adduct with 3,4,5-trimethoxybenzaldehyde, also in toluene under reflux for several hours in the presence of piperidine. Our method for preparing compounds **3a**–**o** in very good yields does not involve toxic solvents like toluene or bases such as piperidine, avoids prolonged heating, and uses sustainable conditions, employing only acetone as a solvent and room-temperature processes.

### 2.2. Biological Activities

#### 2.2.1. Antifungal Activity

The initial antifungal screening revealed that, as reported in Table 2, both Tranilast and its derivatives exhibited significant inhibitory activity against all tested species, with MIC values ranging from 75 to 175 µg/mL. Notably, compounds **3a** and **3b** displayed the strongest antifungal effects, showing the lowest MIC values (75 µg/mL) across all three microorganisms. These findings highlight the encouraging antifungal potential of the parent compound and its structurally related analogues.

#### 2.2.2. Antioxidant Activity

**Figure 4:**
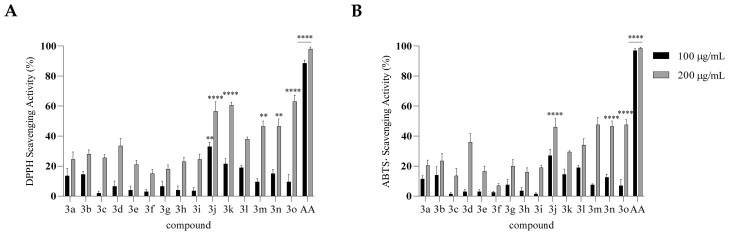
Antioxidant activity of compounds **3a**–**o** assessed using the DPPH (**A**) and ABTS (**B**) assays. Results are shown as % inhibition (mean ± SD). Ascorbic acid (AA) served as a positive control. Asterisks indicate significant differences compared to Tranilast (**2**): ** *p* < 0.01, **** *p* < 0.0001 (Tukey’s test).

The antioxidant activity of the compounds was evaluated using both the DPPH and ABTS radical scavenging assays at concentrations of 100 and 200 µg/mL (Figure 4). Compounds bearing a hydroxyl substituent—particularly derivatives **3m**, **3n**, and **3o**—exhibited the highest radical scavenging capacity in both assays, whereas compounds lacking hydroxyl groups demonstrated only minimal activity. At 200 µg/mL, derivatives **3m**–**o** achieved up to 60% radical scavenging in the DPPH assay and approximately 50% in the ABTS assay, values that were significantly higher than those of Tranilast **3b**. These findings indicate that the introduction of hydroxyl groups substantially enhances the antioxidant potential of these molecules.

#### 2.2.3. In Vitro Cytotoxicity and Antiproliferative Activities

**Figure 5:**
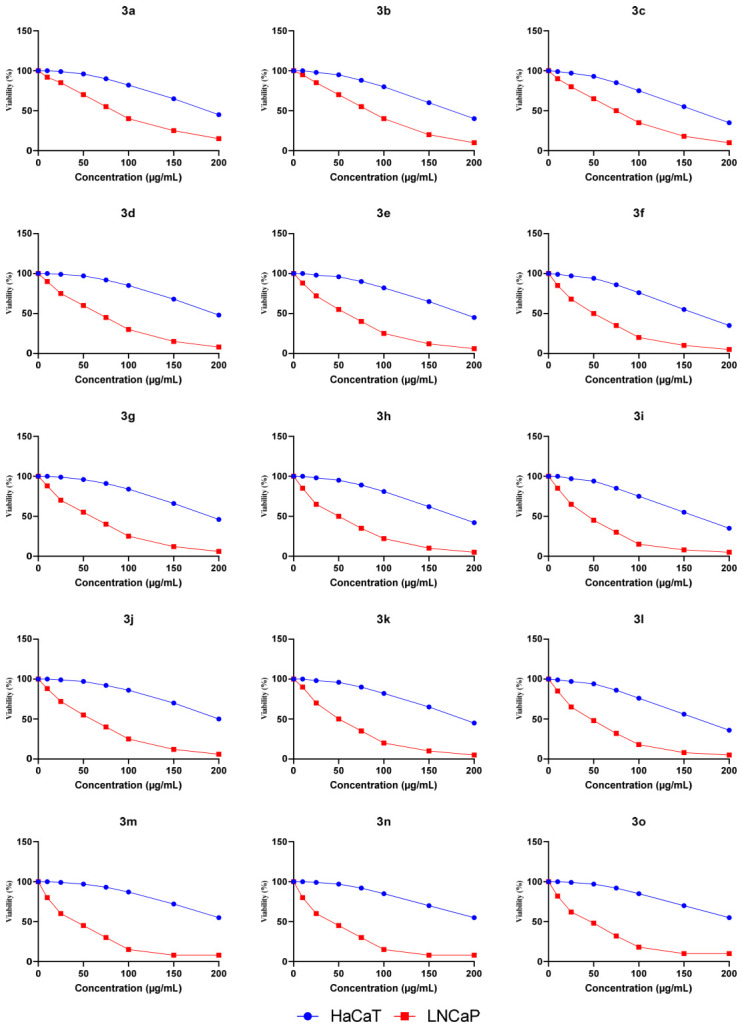
Cytotoxicity of compounds **3a**–**o** on HaCaT and LNCaP cells assessed by MTT assay. Cell viability was measured after 48 h of treatment at different concentrations. Data are shown as the percentage of viable cells compared to untreated control and are presented as the average of three independent experiments.

**Figure 6:**
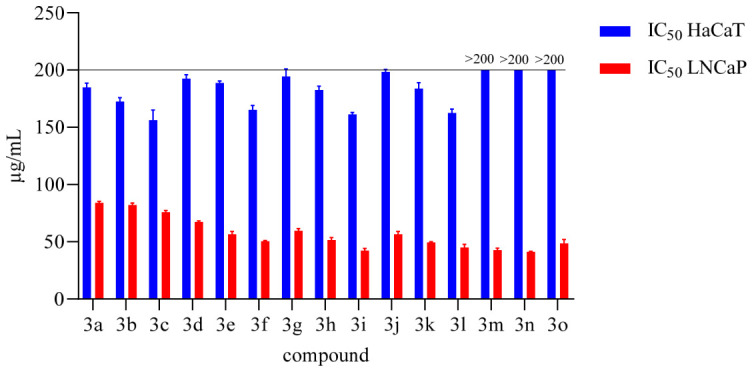
Proliferative effects of compounds **3a**–**o** on human keratinocytes (HaCaT cells) and the human prostate carcinoma cell line (LNCaP). HaCaT cells and LNCaP were treated with various concentrations of the compounds for 48 h. Statistical significance was assessed.

The safety profiles of Tranilast and its derivatives were evaluated by assessing their cytotoxicity in non-tumorigenic HaCaT cells and their antiproliferative effects in LNCaP prostate carcinoma cells using the MTT assay. As shown in Figure 5, the dose–response curves demonstrated a clear differential sensitivity between the two cell lines for all tested compounds. Specifically, the IC_50_ values obtained for HaCaT cells (Figure 6) were generally high (≥150 µg/mL), indicating low cytotoxicity toward normal keratinocytes. In contrast, markedly lower IC_50_ values were observed in LNCaP cells, demonstrating that the malignant cells were more responsive to treatment.

Tranilast (compound **3b**) displayed an IC_50_ of approximately 80 µg/mL in LNCaP cells and around 180 µg/mL in HaCaT cells, consistent with its known moderate antiproliferative effects and favourable tolerability profile in non-cancerous cells. Among the synthesized derivatives, compounds **3i**, **3l**, **3m**, and **3n** exhibited the most indicative activity, with IC_50_ values in LNCaP cells around 45–50 µg/mL, while maintaining IC_50_ values above 150 µg/mL in HaCaT cells. These findings highlight their improved antiproliferative potency and enhanced selectivity relative to the parent compound.

The remaining derivatives displayed moderate to weak activity, with IC_50_ values in LNCaP cells ranging from approximately 55 to 80 µg/mL. Overall, these results suggest that introducing specific structural modifications to Tranilast can enhance its antiproliferative properties while preserving a favourable safety margin toward non-tumor cells.

#### 2.2.4. Anti-Inflammatory Activity

**Figure 7:**
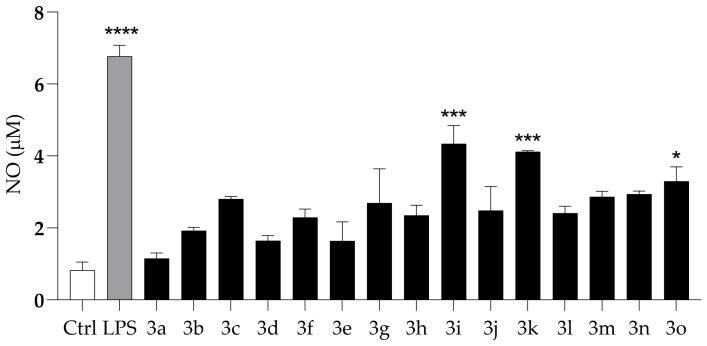
Anti-inflammatory activity of compounds (**3a**–**o**) on LPS-induced NO production in HaCaT cells at a concentration of 100 μg/mL. Asterisks indicate significant differences compared to Tranilast (**3b**): * *p* < 0.05, *** *p* < 0.001, **** *p* < 0.0001 (Dunnett’s test).

To evaluate the potential anti-inflammatory activity of compounds **3a**–**o**, nitric oxide (NO) production was quantified in LPS-stimulated HaCaT cells using the Griess assay. As shown in Figure 7, LPS treatment markedly increased NO levels compared with untreated controls, confirming the responsiveness and reliability of the experimental model. At the tested concentration, none of the compounds induced NO release in the absence of LPS stimulation. Among the tested derivatives, only compounds **3i**, **3k**, and **3o** produced a statistically significant reduction in NO levels compared with compound **3b**. These results indicate a pronounced suppression of NO synthesis and highlight the notable anti-inflammatory potential of these selected derivatives.

#### 2.2.5. Structure–Activity Relationships (SAR)

Antifungal activity (Table 2). Compound **3a** (bearing a single methoxy group at C-4 of the cinnamic portion and lacking –OH groups on the anthranilic ring) shows the lowest MIC values (75 μg/mL) against all three microorganisms. Compounds **3b** (di-OMe at C-3,4) and **3c** (tri-OMe at C-3,4,5) are less active, indicating that increased methoxylation of the cinnamic portion does not enhance antibacterial activity. The introduction of –OH groups on the anthranilic ring does not improve MIC values; in fact, substitutions at C-4′ and C-6′ reduce activity more markedly than those at C-3′ and C-5′.

DPPH Antioxidant Activity (Figure 4A). The presence and position of a hydroxyl group on the anthranilic portion are crucial for radical-scavenging activity: derivatives **3m**–**o** (bearing –OH at C-6′) are the most active (≈60% at 200 μg/mL). Increasing the number of methoxy groups on the cinnamic portion plays a secondary role and enhances the effect only when an –OH group is already present on the anthranilic ring.

ABTS^•+^ Antioxidant Activity (Figure 4B). The profile mirrors that observed for DPPH: the assay is highly sensitive to the presence of an anthranilic –OH group and less influenced by the number of OMe groups on the cinnamic portion. Again, compounds **3m**–**o** (6′-OH) perform best, showing ≈50% inhibition at the highest concentration.

Antiproliferative Activity and Selectivity (LNCaP vs. HaCaT, Figure 5 and Figure 6). Across all series, the LNCaP cell line is more sensitive than HaCaT, indicating good selectivity. Tranilast (**3b**) displays IC_50_ ≈ 80 μg/mL (LNCaP) and ≈180 μg/mL (HaCaT). Introducing hydroxyl groups improves both potency and therapeutic window: compounds **3i**, **3l**, **3m**, and **3n** show IC_50_ ≈ 45–50 μg/mL (LNCaP) and ≥150 μg/mL (HaCaT), with a selectivity index SI > 3. The anthranilic –OH group is therefore essential, with positions C-4′,5′ and 6′ being the most effective. Tri-methoxylation of the cinnamic portion acts as a booster, but only in combination with a phenolic –OH (e.g., compounds **3i**, **3l**–**n**).

Anti-inflammatory Activity (NO production, Figure 7). Only some phenolic compounds significantly reduce NO levels relative to Tranilast (**3b**): compounds **3i** (4′-OH), **3k** (5′-OH), and **3o** (6′-OH). The tri-OMe pattern on the cinnamic portion is not sufficient on its own but enhances the effect in the presence of an –OH in a favorable position, as observed for compound **3o**.

The SAR conclusions indicate that antifungal activity is highest in compound **3a**, which is mono-methoxylated and lacks phenolic hydroxyl groups; antioxidant activity improves with the introduction of an anthranilic –OH, ideally at C-6′, while methoxy groups on the cinnamic portion act as enhancers; antiproliferative activity reaches maximum efficacy when an –OH at C-4′,5′,6′ is combined with multiple methoxy groups on the cinnamic moiety, as seen in lead compounds **3i**, **3l**, **3m**, and **3n**; finally, anti-NO activity is optimal with an –OH at C-4′,5′,6′, whereas the presence of two or three methoxy groups on the cinnamic portion is beneficial only synergistically, as observed in lead compounds **3i**, **3k**, and **3o**.

## 3. Materials and Methods

### 3.1. General Information

Unless explicitly stated otherwise, all reactions were carried out in previously dried solvents. Product identities were verified, whenever feasible, by comparison with reported data, using ^1^H and ^13^C NMR spectroscopy together with high-resolution mass spectrometry. The progress of the reactions was checked by thin-layer chromatography (TLC) on silica gel plates supported on aluminum (Macherey-Nagel, Düren, Germany, Alugram Xtra SIL G/UV254, 0.2 mm). TLC spots were visualized under UV light at 254 nm. NMR spectra were acquired in (CD_3_)_2_SO on a Bruker Avance 400 spectrometer (Billerica, MA, USA, operating at 400 MHz for ^1^H and 101 MHz for ^13^C). Chemical shifts are expressed in parts per million (ppm) relative to the residual solvent signals ((CD_3_)_2_SO: δ 2.50 for ^1^H, δ 39.51 for ^13^C). Signal multiplicities are indicated as follows: s (singlet), d (doublet), dd (doublet of doublets), t (triplet), sept (septet), m (multiplet). MALDI-TOF analyses were conducted using a Voyager-De Pro instrument (PerSeptive Biosystems, Framingham, MA, USA).

### 3.2. General Procedure for the Preparation of N-Methoxycinnamoyl-Hydroxyanthranilic Acids

A solution of methoxycinnamic acids **1a**–**c** (1 mmol) in dry acetone (3 mL) was prepared under magnetic stirring. Triethylamine (1.1 equiv; 1.1 mmol; 152 μL), previously diluted in dry acetone (2 mL), was then added dropwise at room temperature. After several minutes, the resulting pale yellow, homogeneous mixture was cooled in an ice bath. A second dropwise addition was carried out over 15 min, using isobutyl chloroformate (1.1 equiv; 1.1 mmol; 143 μL) dissolved in dry acetone (2 mL). Upon addition, a white solid formed, and the reaction mixture was stirred at room temperature for an additional 20 min. TLC analysis (20% EtOAc in hexane) confirmed complete consumption of the methoxycinnamic acids and formation of the corresponding mixed anhydrides. The mixture was filtered, and the solid residue was washed with dry acetone (2 mL). The combined acetone solution containing the activated cinnamic acids was added dropwise to aromatic amino acids **2a**–**e** (1.05 mmol), followed by triethylamine (1.05 equiv, 1.05 mmol, 146 μL). The resulting suspension was stirred at room temperature for 4 h, during which the amino acids gradually dissolved, yielding a brown solution. TLC analysis (hexane/EtOAc/AcOH/MeOH, 70:30:1:1) confirmed complete consumption of the mixed anhydrides and formation of new products with lower Rf values compared to the starting methoxycinnamic acids. Acetone was evaporated under reduced pressure, and the resulting residue was dissolved in dry diethyl ether (50 mL). The mixture was stirred vigorously at room temperature for 1 h and then filtered. The solid obtained was washed twice with petroleum ether (2 × 10 mL) and dried under a stream of N_2_. In most cases, the crude products were pure by TLC and ^1^H-NMR; if minor impurities remained, the solids were further purified by crystallization from ethyl acetate/hexane. *N*-methoxycinnamoyl -hydroxyanthranilic acids **3a**–**o** were isolated in yields ranging from 63% to 94%. An analytical sample of each of the compounds **3a**–**o** was crystallized for melting point determination.

### 3.3. Antifungal Activity

The following standard ATCC reference strains were used in this study: *Candida albicans* ATCC 90028, *Staphylococcus aureus* ATCC 6538, and *Klebsiella pneumoniae* ATCC 13883. Microbial cultures were grown in Tryptic Soy Broth (TSB), supplemented with 1% *w*/*v* glucose for yeast (VWR Chemicals, Radnor, PA, USA) and without glucose for bacteria, and incubated for 24 h at 37 °C on an orbital rotary shaker. Following incubation, the cells were washed twice with sterile phosphate-buffered saline (PBS) and adjusted to a final concentration of 10^6^ cells/mL in the corresponding culture medium for subsequent assays.

The minimal inhibitory concentrations (MICs) of compounds **3a**–**o** against *S. aureus*, *C. albicans*, and *K. pneumoniae* were determined across a range of concentrations using previously described broth microdilution methods following CLSI guidelines [21,22,23,24]. Amphotericin B (AMP), vancomycin (VAN), and ciprofloxacin (CIP) were used as reference antifungal agents for *C. albicans*, *S. aureus*, and *K. pneumoniae*, respectively. Compounds achieving ≥90% growth inhibition were classified as active.

### 3.4. Antioxidant Activity: DPPH and ABTS Radical Scavenging Assays

The free radical scavenging activity of compounds **3a**–**o** was assessed using both the DPPH (2,2-diphenyl-1-picrylhydrazyl) assay and the ABTS [2,2′-azino-bis(3-ethylbenzothiazoline-6-sulfonic acid)] radical scavenging assay, following the protocol described by Yaermaimaiti et al. [25].

For the DPPH assay, a 0.2 mM DPPH solution in methanol was freshly prepared and protected from light until use. Subsequently, 100 µL of each sample solution was added to individual wells of a 96-well microplate, immediately followed by the addition of 100 µL of the DPPH solution. The plate was incubated for 30 min at room temperature in the dark. Absorbance was then measured at 517 nm using a UV–Vis spectrophotometer (Synergy H4, BioTek Inc., Winooski, VT, USA). The DPPH radical scavenging activity (%) was determined using the following equation:

$$\mathrm{DPPH}\;\mathrm{Scavenging}\;\mathrm{Activity}\;(\%)=\frac{A_{control}-A_{sample}}{A_{control}}\times 100$$

where $A_{control}$ is the absorbance of the DPPH solution without the sample, and $A_{sample}$ is the absorbance in the presence of each compound. Ascorbic acid was used as a positive control.

For the ABTS assay, the ABTS^•+^ solution was diluted with ethanol to achieve an absorbance of 0.70 ± 0.02 at 734 nm. Subsequently, each compound (100 mM) was added to the wells of a 96-well microplate. After incubation at room temperature for 30 min, absorbance was measured at 734 nm.

The ABTS radical scavenging activity (%) was calculated as follows:

$$\mathrm{ABTS}\;\mathrm{Scavenging}\;\mathrm{Activity}\;(\%)=\frac{A_{control}-A_{sample}}{A_{control}}\times 100$$

where $A_{control}$ represents the absorbance of the ABTS^•+^ solution without the sample, and $A_{sample}$ represents the absorbance in the presence of the peptide. Ascorbic Acid was used as a reference antioxidant.

### 3.5. Antitumor Activity

Compounds **3a**–**o** were evaluated for cytotoxicity in HaCaT cells and for antiproliferative effects in the human prostate carcinoma cell line LNCaP using the MTT assay, as previously described [26].

HaCaT and LNCaP cells were cultured in Dulbecco’s Modified Eagle Medium (DMEM; Sigma-Aldrich Co., St. Louis, MO, USA), supplemented with 10% foetal bovine serum, 1% L-glutamine, and 1% penicillin/streptomycin (Sigma-Aldrich). Cells were maintained in a humidified incubator at 37 °C with 5% CO_2_. Upon reaching 70–80% confluence, they were detached using Trypsin/EDTA solution (Sigma-Aldrich) and transferred to new culture flasks. Culture medium was renewed twice weekly, and cell morphology and confluence were monitored daily using an inverted microscope.

For cytotoxicity and antiproliferative assays, cells were seeded in 96-well plates at a density of 2 × 10^4^ cells per well. After 24 h, the medium was replaced with fresh DMEM containing increasing concentrations of the test compounds (10–200 μM). A dose–response curve was generated using an 8-point two-fold serial dilution series. Plates were incubated for 48 h at 37 °C to allow formation of formazan crystals. The MTT-containing medium was then removed, and 100 µL of DMSO was added to each well to solubilize the formazan. Plates were gently shaken for 2 min in the dark, and absorbance was measured at 570 nm using a Varioskan LUX Multimode Microplate Reader (Thermo Fisher Scientific, Waltham, MA, USA). All experiments were performed independently three times, with each experimental condition tested in triplicate.

The half-maximal inhibitory concentration (IC_50_) values were determined by non-linear regression analysis of the dose–response curves using normalized absorbance values as a function of inhibitor concentration. To assess selective cytotoxicity toward tumor cells, the Selectivity Index (SI) was calculated as the ratio of IC_50_ values in HaCaT versus LNCaP cells (SI = IC_50_ HaCaT/IC_50_ LNCaP). An SI value greater than 1 indicates preferential cytotoxicity toward tumor cells relative to non-tumor cells [27].

### 3.6. Anti-Inflammatory Activity Assay

The inhibitory activity of compounds **3a**–**o** on LPS-induced nitric oxide (NO) production was evaluated by quantifying nitrite accumulation in the supernatants of non-tumorigenic human keratinocyte cells (HaCaT). Nitrite levels were measured using a colorimetric assay based on the Griess reagent kit (Invitrogen), following the manufacturer’s protocol. HaCaT cells were seeded at a density of 2.5 × 10^5^ cells/mL in 24-well culture plates and incubated for 18 h. Subsequently, the cells were stimulated with LPS (1 μg/mL) in the presence of each test compound at 100 µg/mL for 24 h. All experiments were performed in triplicate. Absorbance was recorded at 548 nm using a Varioskan LUX Multi-Mode Microplate Reader (Thermo Fisher Scientific, Waltham, MA, USA).

## 4. Conclusions

The notable biological properties and significant impact on human health of many hydroxycinnamic acid derivatives have attracted considerable interest in recent decades. Conjugation, through the formation of an amide bond, between various hydroxycinnamic acids and aromatic amino acids, such as hydroxyanthranilic acids, gives rise to natural avenanthramides, which are well known for their beneficial effects on human health. The synthetic method employed in this study is an established mixed-anhydride procedure that has been widely used for the derivatization of hydroxycinnamic acids. In this work, we apply this known and environmentally friendly approach to the Tranilast scaffold, allowing efficient access to hydroxylated analogues without chromatographic purification. The process is completed in a single coupling step within a few hours and affords higher yields of the desired derivatives using environmentally friendly solvents and reagents. Moreover, it is scalable. The products are obtained in pure form by simple filtration and crystallization, avoiding lengthy and costly chromatographic purification steps. In conclusion, the hydroxylated Tranilast derivatives exhibited measurable antifungal activity, although their potency was considerably lower than that of the reference standard amphotericin B (approximately 600-fold). Compounds **3a** and **3b** showed the lowest MIC values within the series. Hydroxyl substitution improved antioxidant capacity relative to Tranilast, although the activity of the most active derivatives (**3m**–**o**) remained below that of ascorbic acid (approximately half). Cytotoxicity assays confirmed low toxicity toward non-tumor HaCaT cells and moderate antiproliferative activity in LNCaP cells, with compounds **3i**, **3l**, **3m**, and **3n** showing the most favourable selectivity profiles. Furthermore, selected derivatives significantly reduced NO production in LPS-stimulated HaCaT cells, supporting their anti-inflammatory potential. Overall, these findings indicate that structural modifications of Tranilast modulate antifungal, antioxidant, antiproliferative, and anti-inflammatory properties. Although the absolute potency does not match that of reference standards, the series provides useful structure–activity insights for future design of improved analogues.

## Figures and Tables

**Table 1:** Structure and yields of synthesized hydroxylated Tranilast analogs.

No.	Structure	Yield %	No.	Structure	Yield %
**3a**	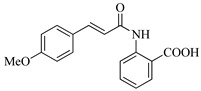	73	**3i**	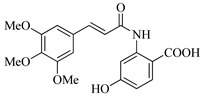	72
**3b**	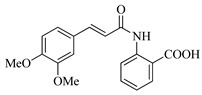	77	**3j**	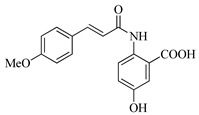	92
**3c**	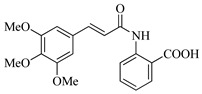	80	**3k**	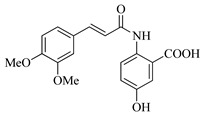	94
**3d**	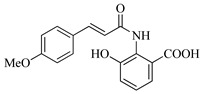	88	**3l**	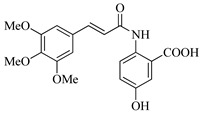	90
**3e**	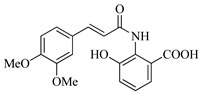	90	**3m**	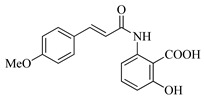	66
**3f**	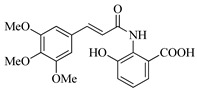	92	**3n**	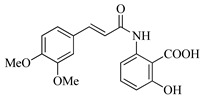	68
**3g**	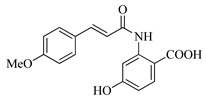	69	**3o**	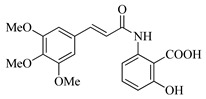	63
**3h**	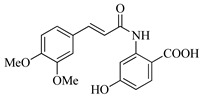	70			

**Table 2:** Evaluation of the minimum inhibitory concentrations (MICs, μg/mL) for compounds **3a**–**o**. The values were obtained from at least three independent experiments. AMP, amphotericin B; CIP, ciprofloxacin; VAN, vancomycin.

No.	*C. albicans*	*K. pneumoniae*	*S. aureus*
**3a**	75 ± 5	75 ± 5	75 ± 5
**3b**	75 ± 5	100 ± 10	100 ± 10
**3c**	100 ± 10	100 ± 10	100 ± 10
**3d**	100 ± 10	100 ± 10	100 ± 10
**3e**	100 ± 10	100 ± 10	100 ± 10
**3f**	100 ± 10	100 ± 10	100 ± 10
**3g**	125 ± 10	120 ± 10	150 ± 15
**3h**	175 ± 15	150 ± 15	200 ± 20
**3i**	100 ± 10	100 ± 10	100 ± 10
**3j**	100 ± 10	125 ± 10	125 ± 10
**3k**	100 ± 10	125 ± 10	150 ± 15
**3l**	150 ± 15	100 ± 10	100 ± 10
**3m**	150 ± 15	150 ± 15	150 ± 15
**3n**	150 ± 15	150 ± 15	125 ± 10
**3o**	150 ± 15	150 ± 15	150 ± 15
AMP	0.125	-	-
CIP	-	0.25	-
VAN	-	-	1

## Data Availability

The original contributions presented in this study are included in the article and Supplementary Materials. Further inquiries can be directed to the corresponding author.

## Supplementary Materials

The following supporting information can be downloaded at https://www.mdpi.com/article/10.3390/molecules31081340/s1. Tables S1–S13. ^1^H, ^13^C and 2D-NMR data for compounds **3a**–**k** and **3m**–**o**, respectively. Figures S1–S24. ^1^H-NMR and ^13^C-NMR for compounds **3a**–**k** and **3m**–**o**, respectively.

## Abbreviations

The following abbreviations are used in this manuscript:

*i*Boc-Cl	Isobutyl chloroformate
TEA	Triethylamine
HCAs	Hydroxycinnamic acids
TGF-β	Transforming Growth Factor-beta
PDGF	Platelet-Derived Growth Factor
DPPH	2,2-Difenil-1-Picrylhydrazil
ABTS	2,2′-Azino-bis(3-ethylbenzothiazoline-6-sulfonic acid)
LNCaP	Lymph Node Carcinoma of the Prostate
LPS	Lipopolysaccharide
HaCaT	Human Adult keratinocytes containing Spontaneous Transformations
MCF-7	Michigan Cancer Foundation-7
HepG2	Immortal human hepatocellular carcinoma cell line
TLC	Thin-Layer Chromatography
