# Supplementary material for: Sustainable Synthesis of Novel Hydroxylated Tranilast Analogues and Their Bioactivities
Source: Molecules. 2026 Apr 19;31(8):1340. doi: 10.3390/molecules31081340 (PMC13118400; doi:10.3390/molecules31081340)
Supplement: Supplementary file 1 [file molecules-31-01340-s001.zip › molecules-4212719-supplementary.pdf]

Supporting Information

Sustainable Synthesis of Novel Hydroxylated Tranilast Analogues and Their Bioactivities

Angela Maione[1†], Marianna Imparato[1†], Luigi Cirillo[2], Marco Guida[1], Emilia Galdiero[1], Armando Zarrelli[3,*], and Luigi Longobardo[3]

[1] Department of Biology, University of Naples Federico II, 80126 Naples, Italy;
[2] Division of Urology, Asl Napoli3 Sud, 'San Leonardo' Hospital, 80053 Castellammare di Stabia, Italy; cirilloluigi22@gmail.com
[3] Department of Chemical Science, University of Napoli Federico II, Via Cinthia 4, 80126 Napoli, Italy; luilongo@unina.it
* Correspondence: zarrelli@unina.it

[†]These authors contributed equally to this work

Supporting Information document collects the spectroscopic data relating to the structures described in the main manuscript. For each compound, tables containing the NMR data are provided, along with the original spectra used for their assignment. All NMR spectra were recorded in $(CD_3)_2SO$ on a Bruker Avance 400 spectrometer operating at 400 MHz for ^{1}H and 101 MHz for ^{13}C. For ^{1}H, ^{13}C chemical shifts are presented in δ-scale as ppm (parts per million) with the residual solvent peak as the reference (2.50 ppm for ^{1}H and 39.51 ppm for ^{13}C). The description of the multiplicity designations is as follows: s = singlet, d = doublet, dd = doublet of doublets, t = triplet, sept = septet, m = multiplet. MALDI-TOF mass spectrometric analyses were conducted on a Voyager-De Pro MALDI mass-spectrometer (PerSeptive Biosystems, Framingham, MA, USA). The melting points of all obtained products were determined using a Gallenkamp MFB 595 melting point apparatus (Gallenkamp, Loughborough, UK).

The information provided is intended to ensure complete characterization of the studied molecules and to support the reproducibility of the results.

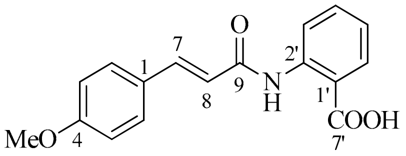

3a: (*E*)-2-(3-(4-Methoxyphenyl)acrylamido)benzoic acid. Beige powder; mp 193-195 °C. ^{1}H- and ^{13}C-NMR: see Zarrelli et Longobardo [1].

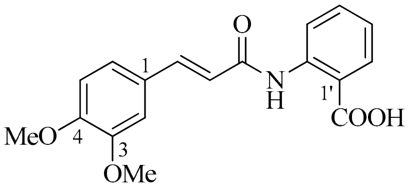

3b: (*E*)-2-(3-(3,4-Dimethoxyphenyl)acrylamido)benzoic acid. Beige powder; mp 166-168 °C. ^{1}H- and ^{13}C-NMR: see Zarrelli et Longobardo [1].

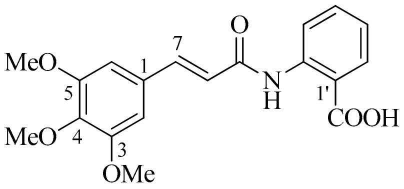

3c: (*E*)-3-(3,4,5-Trimethoxyphenyl)acrylamido)benzoic acid. Beige powder; mp 168-170 °C. ^{1}H- and ^{13}C-NMR: see Table S1. MS-TOF (negative ions): $[M]^-$ calculated for $C_{19}H_{19}NO_6$: *m/z* 357.1212; found 356.3575 $[M - H]^-$ (82%).

Table S1. ^{1}H, ^{13}C and 2D-NMR data of 3c in $(CD_3)_2SO$.

Position	Residue	$^{13}C^{a}$	$^{1}H^{a}$, multiplicity (J in Hz)	^{1}H-^{1}H COSY	^{1}H-^{13}C HMBC
1	C	130.21			
2/6	CH	105.53	7.02, s		140.83, 138.97, 105.53
3/5	C	153.07			
4	C	138.97			
7	CH	140.83	7.54, d (15.6)	6.77	163.55, 122.64, 105.53
8	CH	122.64	6.77, d (15.6)	7.54	163.55, 140.83, 130.21
9	C	163.55			
1'	C	122.12			
2'	C	140.39			
3'	CH	119.08	8.64, d (8.2)	7.42	122.12, 121.85
4'	CH	131.36*	7.42, t (7.1)	8.64, 7.04	140.39, 131.29*
5'	CH	121.85	7.04, t (7.5)	8.07, 7.42	122.12, 119.08
6'	CH	131.29*	8.07, d (8.0)	7.04	172.00, 140.39, 131.36*
7'	C	172.00			
3,5-OCH_3	CH_3	55.99	3.83, s		153.07
4-OCH_3	CH_3	60.08	3.70, s		138.97

[a]Chemical shifts in ppm. * Interchangeable values.

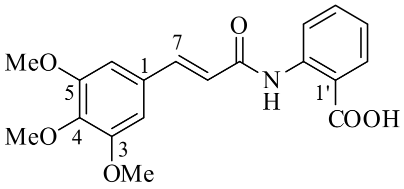

Figure S1. ^{1}H-NMR of compound 3c in $(CD_3)_2SO$

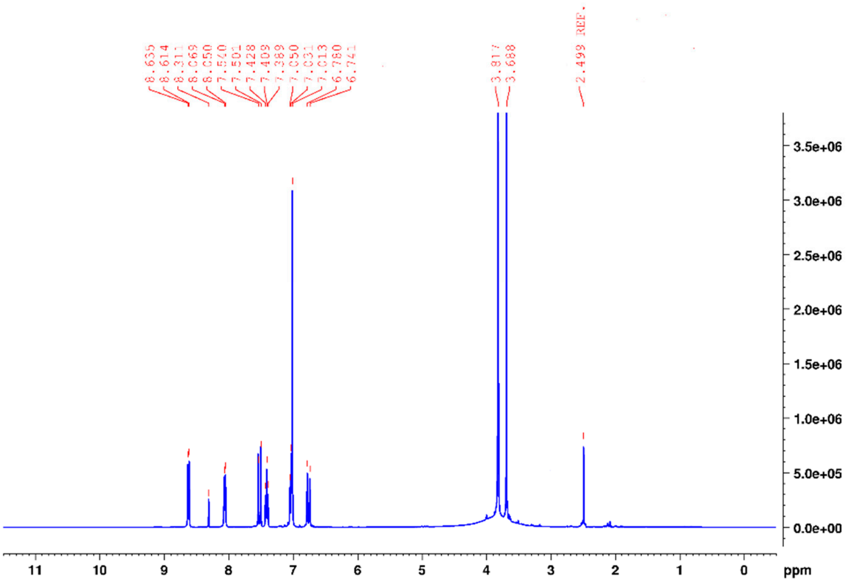

Figure S2. ^{13}C -NMR of compound 3c in $(CD_3)_2SO$

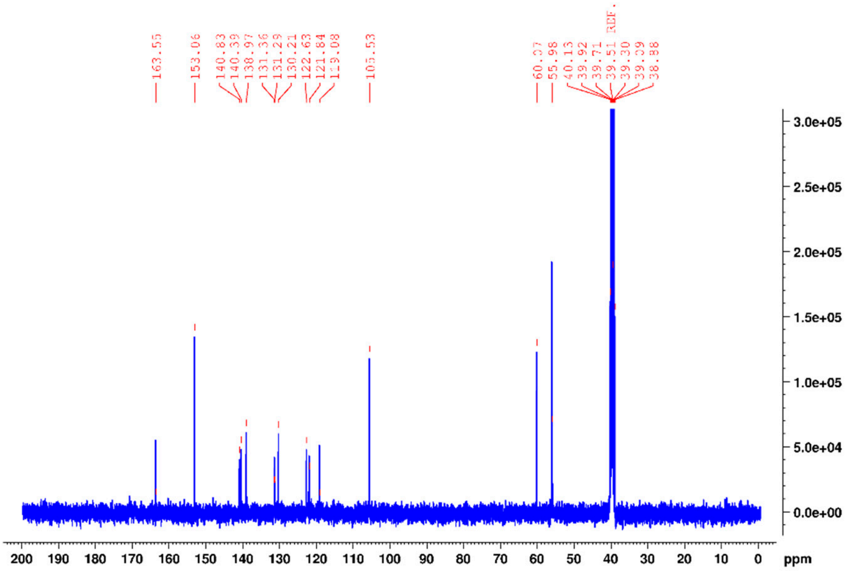

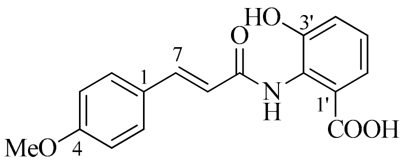

3d: (*E*)-3-Hydroxy-2-(3-(4-methoxyphenyl)acrylamido)benzoic acid. Beige powder; mp 214-216 °C. ^{1}H- and ^{13}C-NMR: see Table S2. MS-TOF (negative ions): $[M]^-$ calculated for $C_{17}H_{15}NO_5$: *m/z* 313.0950; found 312.3045 $[M - H]^-$ (60%).

Table S2. ^{1}H, ^{13}C and 2D-NMR data of 3d in $(CD_3)_2SO$.

Position	Residue	$^{13}C^a$	$^{1}H^a$, multiplicity (J in Hz)	^{1}H-^{1}H COSY	^{1}H-^{13}C HMBC
1	C	127.27			
2/6	CH	129.57	7.61, d (8.8)	7.01	160.75, 140.54, 129.57
3/5	CH	114.52	7.01, d (8.8)	7.61	127.27, 114.52
4	C	160.75			
7	CH	140.54	7.53, d (15.8)	6.85	164.91,129.57, 119.17
8	CH	119.17	6.85, d (15.8)	7.53	164.91, 127.27
9	C	164.91			
1’	C	127.77			
2’	C	125.72			
3’	C	151.06			
4’	CH	120.23	7.09, d (7.9)	7.14	125.72, 120.96
5’	CH	125.29	7.14, t (7.9)	7.30, 7.09	151.06, 127.77
6’	CH	120.96	7.30, d (7.8)	7.14	168.44, 125.72, 120.23
7’	C	168.44			
4-OCH_3	CH_3	55.35	3.82, s		160.75

[a]Chemical shifts in ppm.

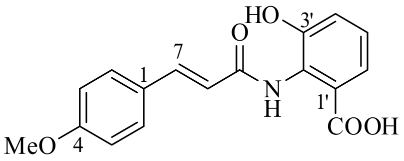

Figure S3. ^{1}H-NMR of compound 3d in $(CD_3)_2SO$

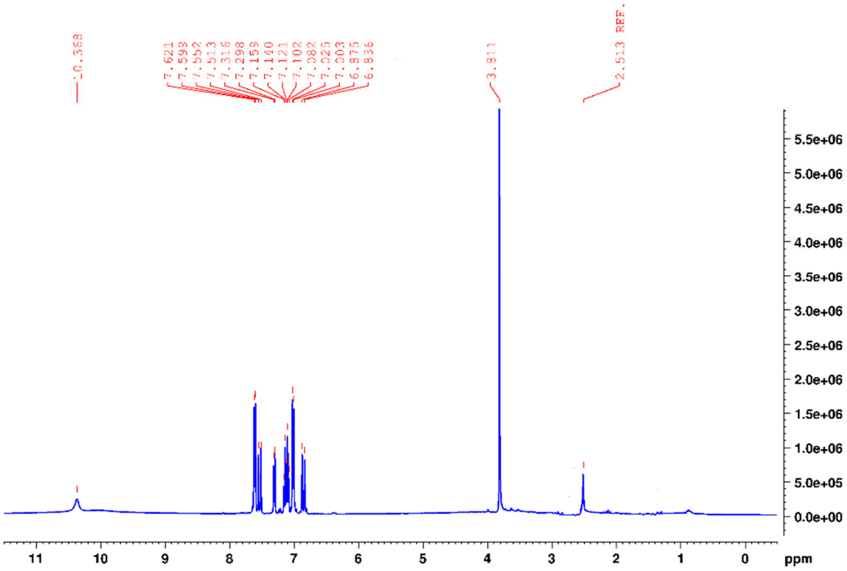

Figure S4. ^{13}C-NMR of compound 3d in $(CD_3)_2SO$

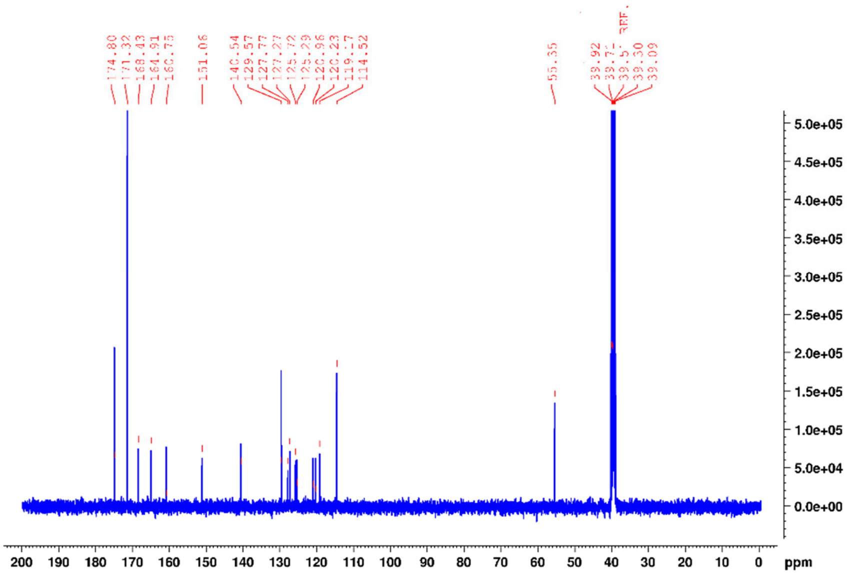

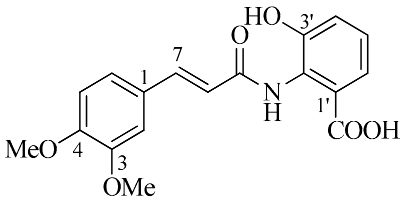

3e: (*E*)-2-(3-(3,4-Dimethoxyphenyl)acrylamido)-3-hydroxybenzoic acid. Beige powder; mp 208-210 °C. ^{1}H- and ^{13}C-NMR: see Table S3. MS-TOF (negative ions): $[M]^-$ calculated for $C_{18}H_{17}NO_6$: *m/z* 343.1056; found 342.3304 $[M - H]^-$ (65%).

Table S3. ^{1}H, ^{13}C and 2D-NMR data of 3e in $(CD_3)_2SO$.

Position	Residue	$^{13}C^a$	$^{1}H^a$, multiplicity (J in Hz)	^{1}H-^{1}H COSY	^{1}H-^{13}C HMBC
1	C	127.48			
2	CH	110.11	7.27, s		150.50, 140.81, 122.03
3	C	148.96			
4	C	150.50			
5	CH	111.74	7.02, d (8.2)	7.20	148.96, 127.48
6	CH	122.03	7.20, d (8.2)	7.02	150.50, 140.81, 110.11
7	CH	140.81	7.52, d (15.7)	6.93	164.81, 122.03, 110.11
8	CH	119.38	6.93, d (15.7)	7.52	164.81, 140.81, 127.48
9	C	164.81			
1’	C	127.64			
2’	C	125.00*			
3’	C	151.14			
4’	CH	120.11	7.11, d (8.0)	7.15	125.00*, 120.79
5’	CH	125.73*	7.15, t (8.0)	7.29, 7.11	151.14, 127.64
6’	CH	120.79	7.29, d (7.9)	7.15	168.30, 125.00*, 120.11
7’	C	168.30			
3-OCH_3	CH_3	55.56	3.84, s		148.96
4-OCH_3	CH_3	55.47	3.81, s		150.50

[a]Chemical shifts in ppm. * Interchangeable values.

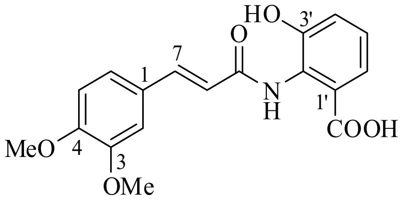

Figure S5. ^{1}H-NMR of compound 3e in $(CD_3)_2SO$

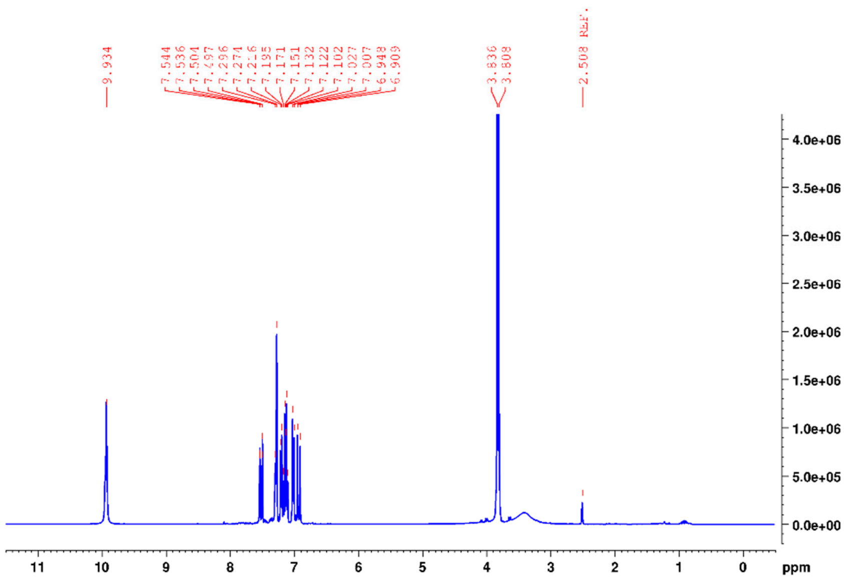

Figure S6. ^{13}C-NMR of compound 3e in $(CD_3)_2SO$

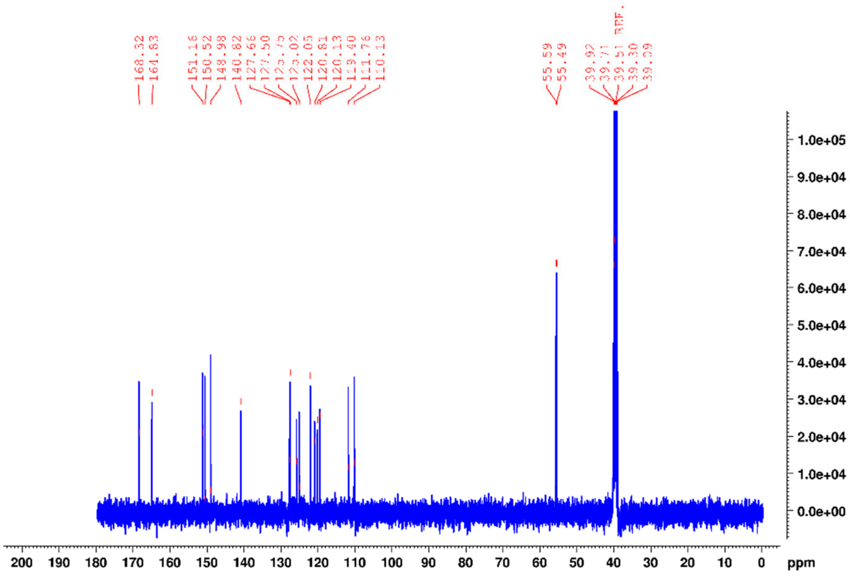

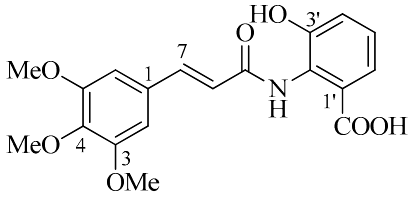

3f: (*E*)-3-Hydroxy-2-(3-(3,4,5-trimethoxyphenyl)acrylamido)benzoic acid. Beige powder; mp 211-213 °C. ^{1}H- and ^{13}C-NMR: see Table S4. MS-TOF (negative ions): $[M]^-$ calculated for $C_{19}H_{19}NO_7$: *m/z* 373.1162; found 373.3564 $[M - H]^-$ (61%).

Table S4. ^{1}H, ^{13}C and 2D-NMR data of 3f in $(CD_3)_2SO$.

Position	Residue	$^{13}C^a$	$^1H^a$, multiplicity (J in Hz)	^{1}H-^{1}H COSY	^{1}H-^{13}C HMBC
1	C	130.35			
2/6	CH	105.29	7.00, s		140.71, 139.00, 105.29
3/5	C	153.14			
4	C	139.00			
7	CH	140.71	7.51, d (15.6)	7.01	164.49, 105.29
8	CH	121.20	7.01, d (15.6)	7.51	164.49, 140.71, 130.35
9	C	164.49			
1’	C	127.92			
2’	C	125.80			
3’	C	151.20			
4’	CH	119.94	7.11, d (7.9)	7.15	125.80, 120.71
5’	CH	124.79	7.15, t (8.0)	7.28, 7.11	151.20, 127.92
6’	CH	120.71	7.28, d (7.5)	7.15	168.26, 125.80, 119.94
7’	C	168.26			
3,5-OCH$_3$	CH$_3$	55.93	3.85, s		153.14
4-OCH$_3$	CH$_3$	60.13	3.71, s		139.00

[a]Chemical shifts in ppm.

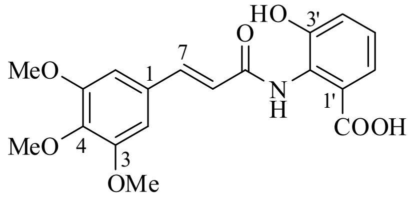

Figure S7. ^{1}H-NMR of compound 3f in $(CD_3)_2SO$

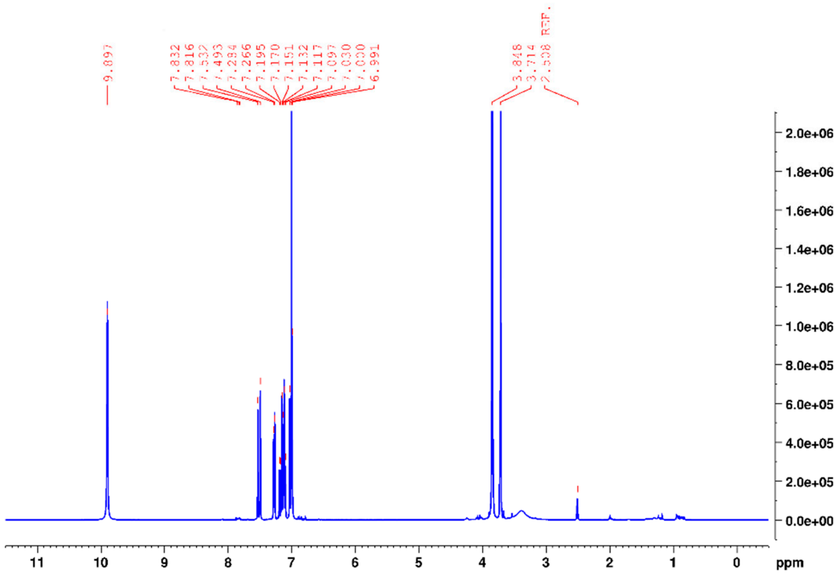

Figure S8. ^{13}C-NMR of compound 3f in $(CD_3)_2SO$

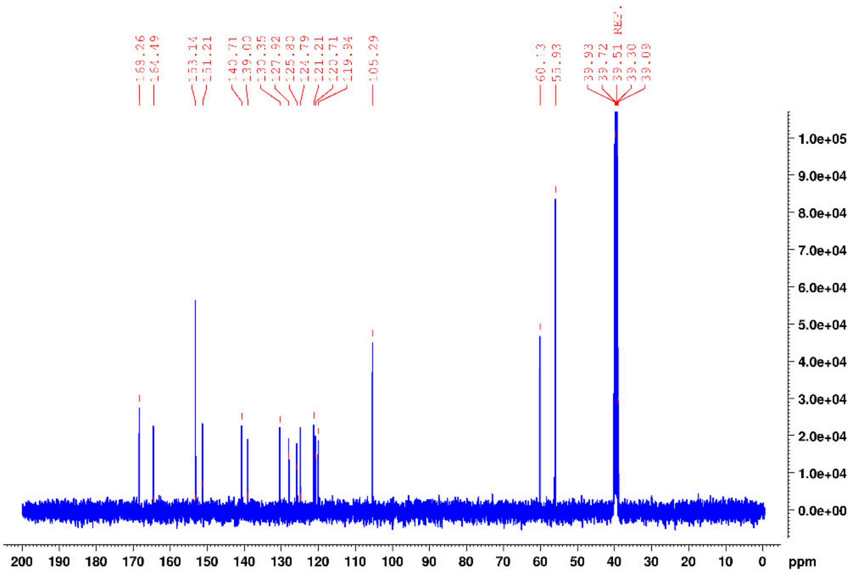

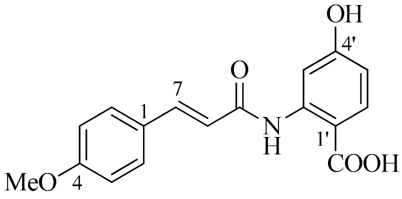

3g: (*E*)-4-Hydroxy-2-(3-(4-methoxyphenyl)acrylamido)benzoic acid. Beige powder; mp 210-211 °C. ^{1}H- and ^{13}C-NMR: see Table S5. MS-TOF (negative ions): $[M]^-$ calculated for $C_{17}H_{15}NO_5$: *m/z* 313.0950; found 312.3043 $[M - H]^-$ (61%).

Table S5. ^{1}H, ^{13}C and 2D-NMR data of 3g in $(CD_3)_2SO$.

Position	Residue	$^{13}C^a$	$^{1}H^a$, multiplicity (J in Hz)	^{1}H-^{1}H COSY	^{1}H-^{13}C HMBC
1	C	127.07			
2/6	CH	129.69	7.67, d (8.8)	6.99	160.66, 140.39, 129.69
3/5	CH	114.33	6.99, d (8.8)	7.67	127.07, 114.33
4	C	160.66			
7	CH	140.39	7.55, d (15.6)	6.61	163.79, 129.69, 120.42
8	CH	120.42	6.61, d (15.6)	7.55	163.79, 140.39, 127.07
9	C	163.79			
1’	C	109.51			
2’	C	142.92			
3’	CH	105.76	8.19, s		109.59, 109.51
4’	C	161.27			
5’	CH	109.59	6.48, d (8.3)	7.87	109.51, 105.76
6’	CH	133.03	7.87, d (8.3)	6.48	169.92, 161.27, 142.92
7’	C	169.92			
4-OCH_3	CH_3	55.27	3.82, s		160.66

[a]Chemical shifts in ppm.

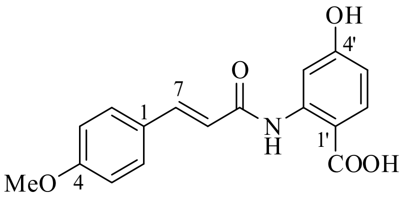

Figure S9. ^{1}H-NMR of compound 3g in $(CD_3)_2SO$

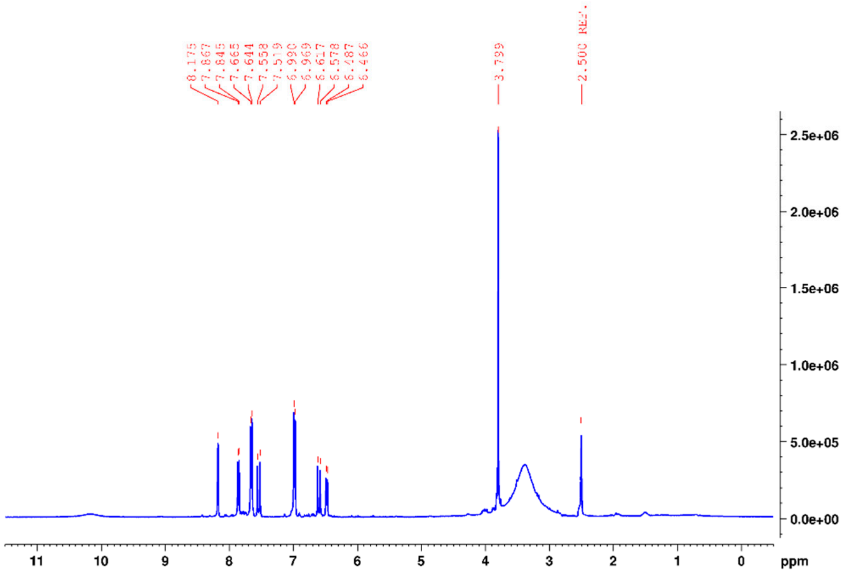

Figure S10. ^{13}C-NMR of compound 3g in $(CD_3)_2SO$

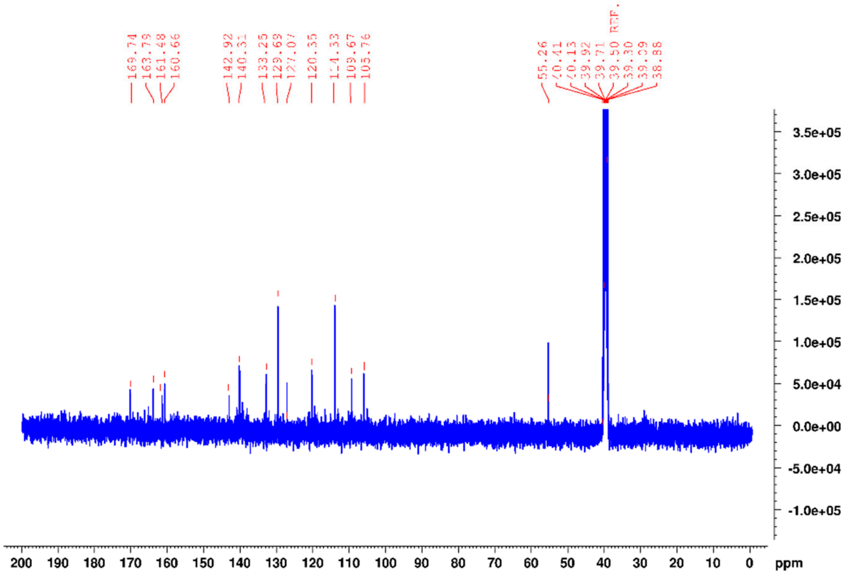

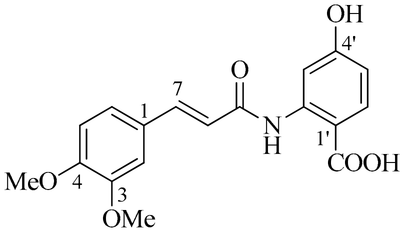

3h: (*E*)-2-(3-(3,4-Dimethoxyphenyl)acrylamido)-4-hydroxybenzoic acid. Beige powder; mp 215-217 °C. ^{1}H- and ^{13}C-NMR: see Table S6. MS-TOF (negative ions): [M]$^-$ calculated for $C_{18}H_{17}NO_6$: *m/z* 343.1056; found 342.3308 [M - H]$^-$ (43%).

Table S6. ^{1}H, ^{13}C and 2D-NMR data of 3h in $(CD_3)_2SO$.

Position	Residue	$^{13}C^a$	$^1H^a$, multiplicity (J in Hz)	^{1}H-^{1}H COSY	^{1}H-^{13}C HMBC
1	C	127.21			
2	CH	110.51	7.38, s		150.66, 141.50, 122.55
3	C	149.04			
4	C	150.66			
5	CH	111.53	7.00, d (8.3)	7.25	149.04, 127.21
6	CH	122.55	7.25, d (8.3)	7.00	150.66, 141.50, 110.51
7	CH	141.50	7.56, d (15.4)	6.73	164.14, 122.55, 110.51
8	CH	120.05	6.73, d (15.4)	7.56	164.14, 127.21
9	C	164.14			
1’	C	106.97			
2’	C	143.36			
3’	CH	106.16	8.24, d (2.1)	6.54	110.07, 106.97
4’	C	162.49			
5’	CH	110.07	6.54, dd (8.5; 2.1)	8.24, 7.89	106.97, 106.16
6’	CH	133.19	7.89, d (8.5)	6.54	169.61. 162.49, 143.36
7’	C	169.61			
3-OCH_3	CH_3	55.67	3.84, s		149.04
4-OCH_3	CH_3	55.53	3.81, s		150.66

[a]Chemical shifts in ppm.

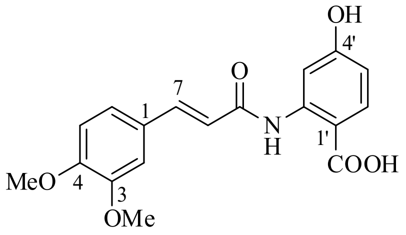

Figure S11. ^{1}H-NMR of compound 3h in $(CD_3)_2SO$

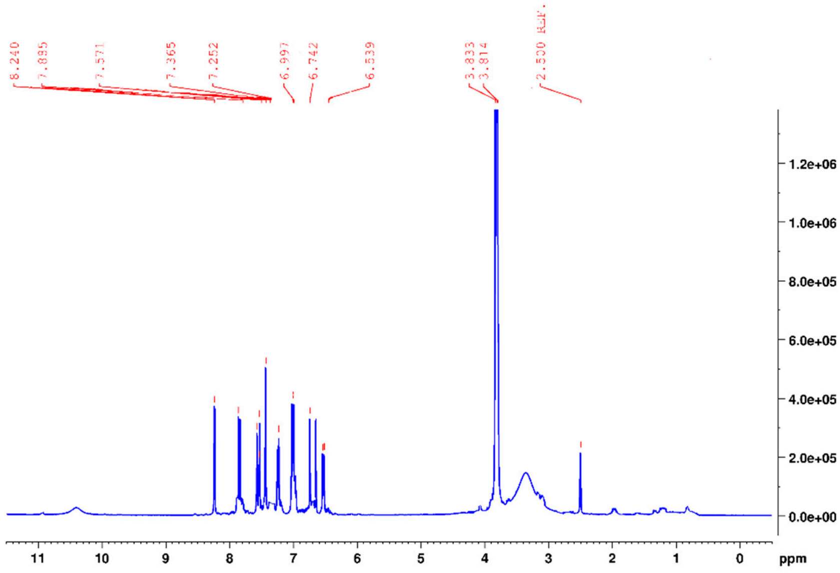

Figure S12. ^{13}C-NMR of compound 3h in $(CD_3)_2SO$

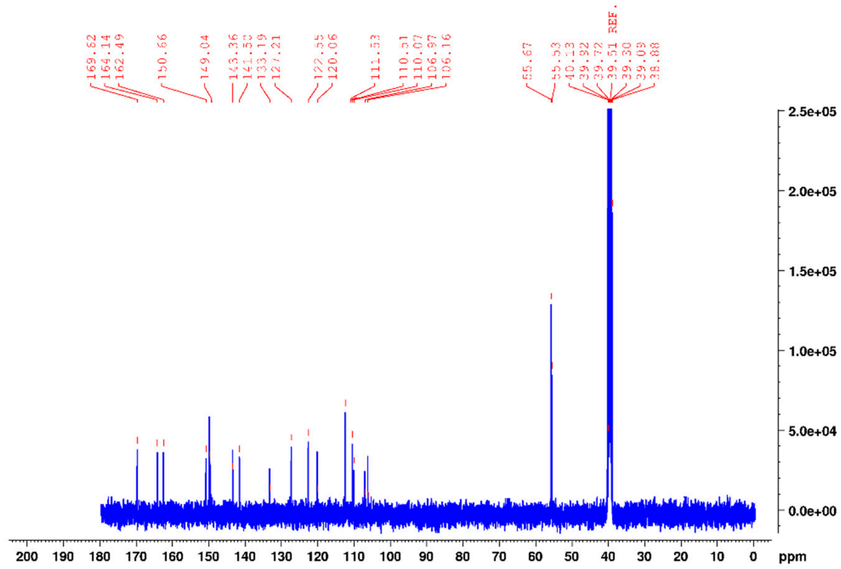

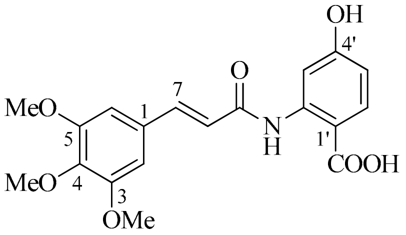

3i: (*E*)-4-Hydroxy-2-(3-(3,4,5-trimethoxyphenyl)acrylamido)benzoic acid. Beige powder; mp 212-214 °C. ^{1}H- and ^{13}C-NMR: see Table S7. MS-TOF (negative ions): $[M]^-$ calculated for $C_{19}H_{19}NO_7$: *m/z* 373.1162; found 372.3569 $[M - H]^-$ (68%).

Table S7. ^{1}H, ^{13}C and 2D-NMR data of 3i in $(CD_3)_2SO$.

Position	Residue	$^{13}C^a$	$^{1}H^a$, multiplicity (J in Hz)	^{1}H-^{1}H COSY	^{1}H-^{13}C HMBC
1	C	130.05			
2/6	CH	105.85	7.08, s		141.73, 139.20, 105.85
3/5	C	153.13			
4	C	139.20			
7	CH	141.73	7.57, d (15.4)	6.85	164.05, 121.78, 105.85
8	CH	121.78	6.85, d (15.4)	7.57	164.05, 141.73, 130.05
9	C	164.05			
1'	C	106.93			
2'	C	143.36			
3'	CH	106.30	8.26, d (2.1)	6.55	110.26, 106.93
4'	C	162.62			
5'	CH	110.26	6.55, dd (8.7; 2.1)	8.26, 7.89	106.93, 106.30
6'	CH	133.29	7.89, d (8.7)	6.55	169.65, 162.62, 143.36
7'	C	169.65			
3,5-OCH_3	CH_3	56.07	3.85, s		153.13
4-OCH_3	CH_3	60.11	3.71, s		139.20

[a]Chemical shifts in ppm

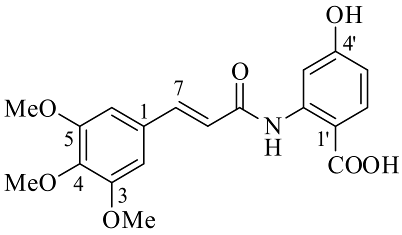

Figure S13. ¹H-NMR of compound 3i in $(CD_3)_2SO$

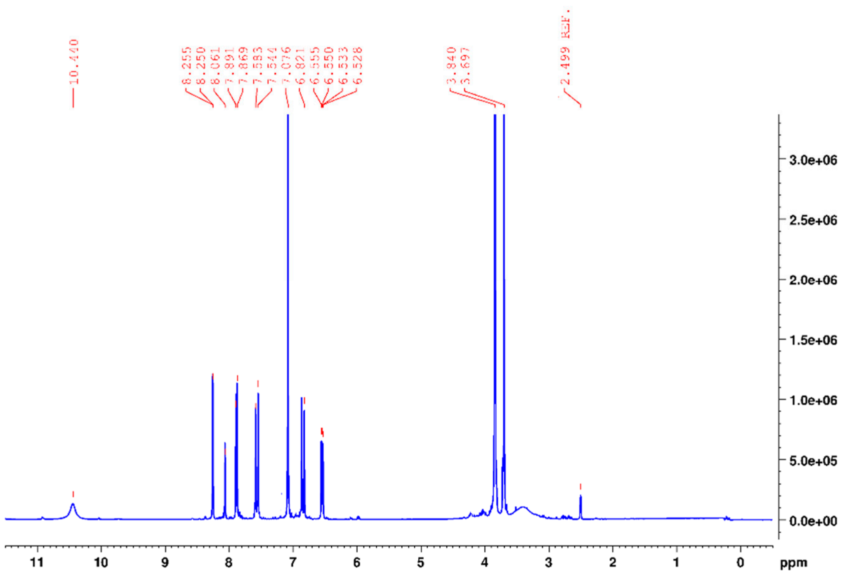

Figure S14. ¹³C-NMR of compound 3i in $(CD_3)_2SO$

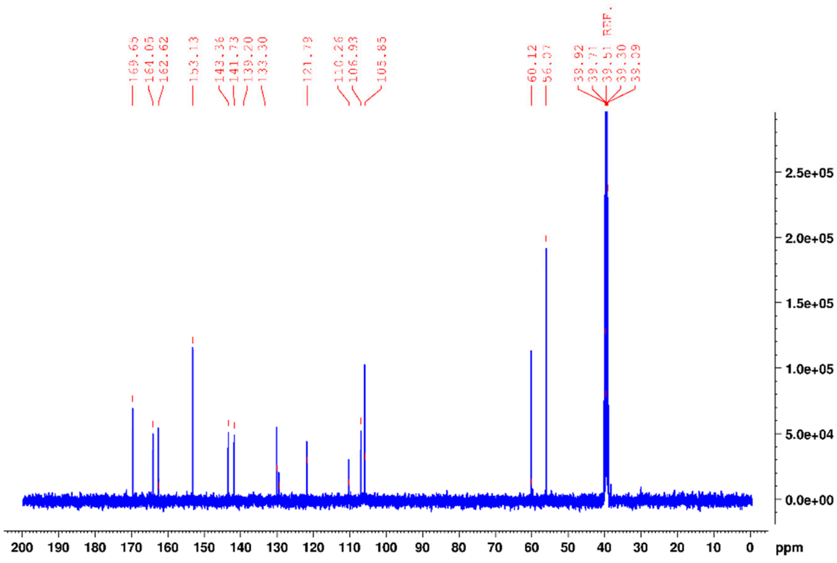

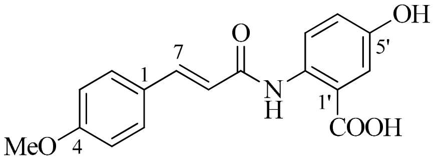

3j: (*E*)-5-Hydroxy-2-(3-(4-methoxyphenyl)acrylamido)benzoic acid. Beige powder; mp 220-222 °C. ^{1}H- and ^{13}C-NMR: see Table S8. MS-TOF (negative ions): $[M]^-$ calculated for $C_{17}H_{15}NO_5$: *m/z* 313.0950; found 312.3044 $[M - H]^-$ (55%).

Table S8. ^{1}H, ^{13}C and 2D-NMR data of 3j in $(CD_3)_2SO$.

Position	Residue	$^{13}C^a$	$^{1}H^a$, multiplicity (J in Hz)	^{1}H-^{1}H COSY	^{1}H-^{13}C HMBC
1	C	127.20			
2/6	CH	129.73	7.63, d (8.7)	6.96	160.70, 140.34, 129.73
3/5	CH	114.38	6.96, d (8.7)	7.63	127.20, 114.38
4	C	160.70			
7	CH	140.34	7.53, d (15.6)	6.67	163.60, 129.73
8	CH	120.08	6.67, d (15.6)	7.53	163.60, 140.34, 127.20
9	C	163.60			
1’	C	118.58			
2’	C	132.90			
3’	CH	122.53	8.37, d (9.2)	7.04	152.62, 118.58
4’	CH	120.93	7.04, dd (9.2; 3.1)	8.37, 7.40	132.90, 116.60
5’	C	152.62			
6’	CH	116.60	7.40, d (3.1)	7.04	169.21, 132.90, 120.93
7’	C	169.21			
4-OCH_3	CH_3	55.29	3.79, s		160.70

[a]Chemical shifts in ppm.

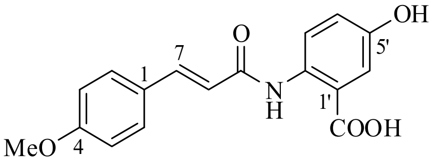

Figure 15. ^{1}H-NMR of compound 3j in $(CD_3)_2SO$

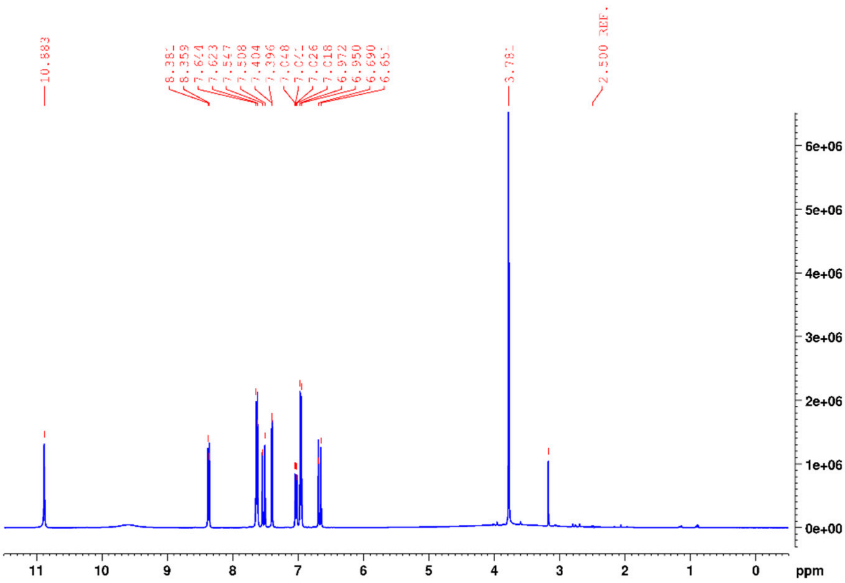

Figure S16. ^{13}C-NMR of compound 3j in $(CD_3)_2SO$

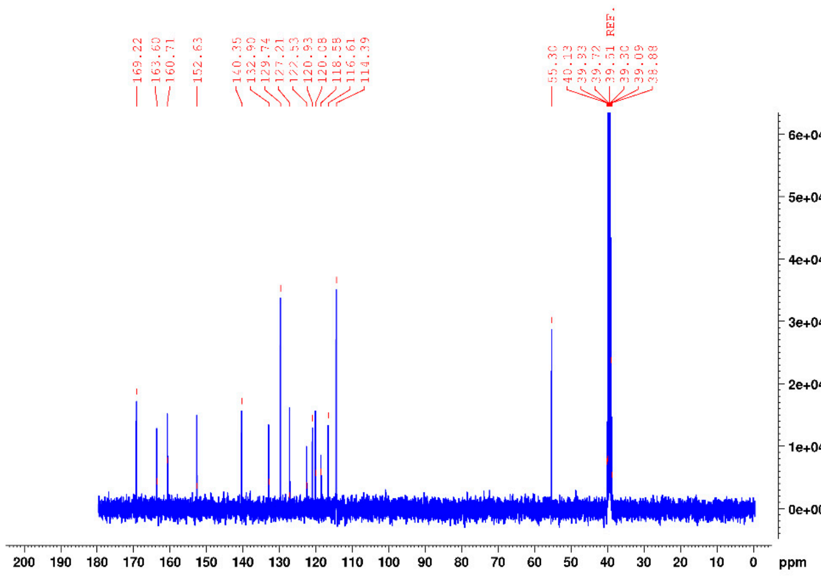

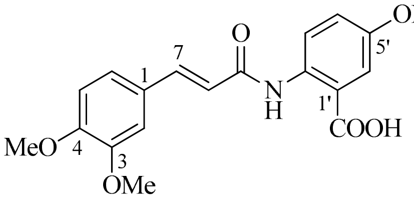

3k: (*E*)-2-(3-(3,4-Dimethoxyphenyl)acrylamido)-5-hydroxybenzoic acid. Beige powder; mp 218-220 °C. ^{1}H- and ^{13}C-NMR: see Table S9. MS-TOF (negative ions): $[M]^{-}$ calculated for $C_{18}H_{17}NO_6$: *m/z* 343.1056; found 342.3306 $[M - H]^{-}$ (62%).

Table S9. ^{1}H, ^{13}C and 2D-NMR data of 3k in $(CD_3)_2SO$.

Position	Residue	$^{13}C^{a}$	$^{1}H^{a}$, multiplicity (J in Hz)	^{1}H-^{1}H COSY	^{1}H-^{13}C HMBC
1	C	126.88			
2	CH	109.88	7.35, d (1.8)	7.23	149.97, 140.21, 121.79
3	C	148.45			
4	C	149.97			
5	CH	111.11	6.99, d (8.4)	7.23	148.45, 126.88
6	CH	121.79	7.23, dd (8.4; 1.9)	7.35, 6.99	149.97, 140.21, 109.88
7	CH	140.21	7.51, d (15.4)	6.75	163.06, 121.79, 119.67, 109.88
8	CH	119.67	6.75, d (15.4)	7.51	163.06, 126.88
9	C	163.06			
1’	C	117.96			
2’	C	132.34			
3’	CH	121.91	8.38, d (9.0)	7.03	151.99, 117.96
4’	CH	120.31	7.03, dd (9.0; 3.0)	8.38, 7.40	132.34, 115.99
5’	C	151.99			
6’	CH	115.99	7.40, d (3.0)	7.03	168.58, 132.34, 120.31
7’	C	168.58			
3-OCH_3	CH_3	55.10	3.84, s		148.45
4-OCH_3	CH_3	55.03	3.81, s		149.97

[a]Chemical shifts in ppm.

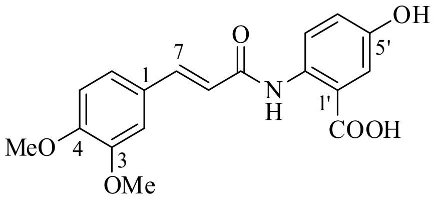

Figure S17. ^{1}H-NMR of compound 3k in $(CD_3)_2SO$

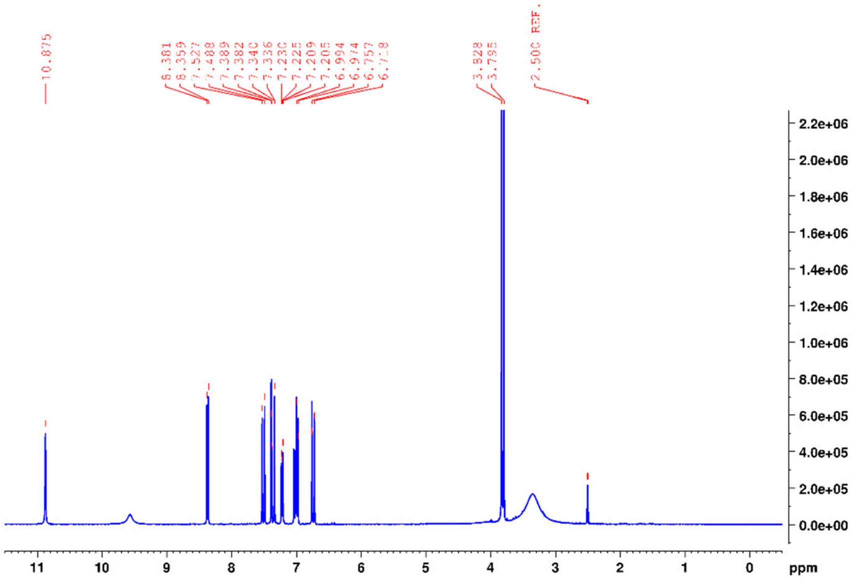

Figure S18. ^{13}C-NMR of compound 3k in $(CD_3)_2SO$

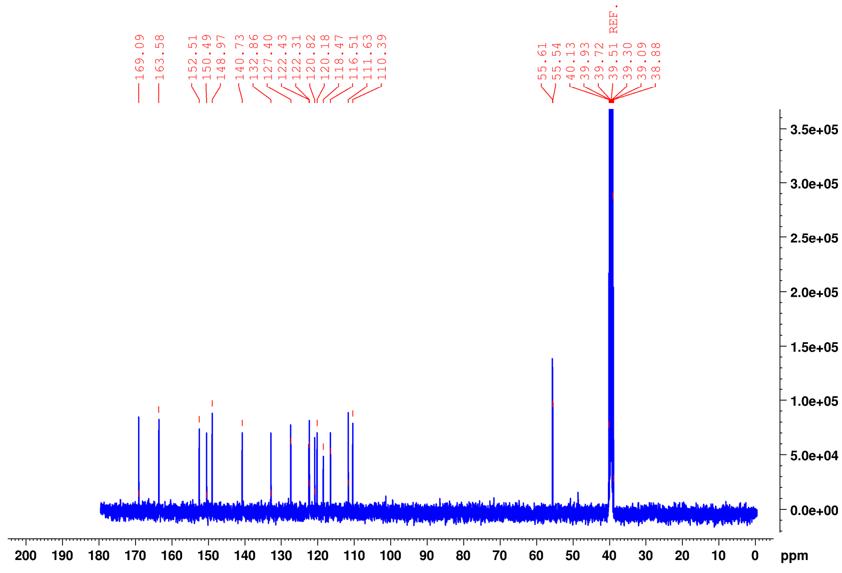

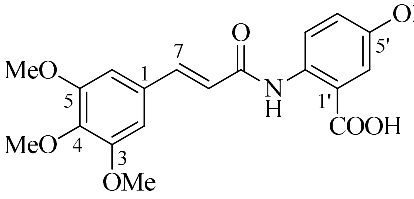

3l: (*E*)-5-Hydroxy-2-(3-(3,4,5-trimethoxyphenyl)acrylamido)benzoic acid. Beige powder; mp 223-225 °C. ^{1}H- and ^{13}C-NMR: see Zarrelli et Longobardo [2].

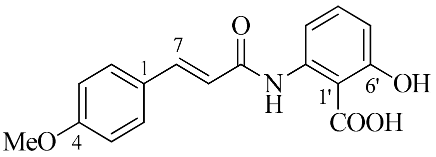

3m: ((*E*)-2-Hydroxy-6-(3-(4-methoxyphenyl)acrylamido)benzoic acid. Beige powder; mp 219-221 °C. ^{1}H- and ^{13}C-NMR: see Table S10. MS-TOF (negative ions): $[M]^{-}$ calculated for $C_{17}H_{15}NO_5$: *m/z* 313.0950; found 312.3044 $[M - H]^{-}$ (54%).

Table S10. ^{1}H, ^{13}C and 2D-NMR data of 3m in $(CD_3)_2SO$.

Position	Residue	$^{13}C^{a}$	$^{1}H^{a}$, multiplicity (J in Hz)	^{1}H-^{1}H COSY	^{1}H-^{13}C HMBC
1	C	127.17			
2/6	CH	129.71	7.63, d (8.7)	7.00	160.75, 140.57, 129.71
3/5	CH	114.44	7.00, d (8.7)	7.63	127.17, 114.44
4	C	160.75			
7	CH	140.57	7.54, d (15.7)	6.70	163.92, 120.02, 129.71
8	CH	120.02	6.70, d (15.7)	7.54	163.92, 127.17
9	C	163.92			
1’	C	106.71			
2’	C	139.91			
3’	CH	112.30	7.79, d (8.3)	7.36	112.06, 106.71
4’	CH	133.41	7.36, t (8.3)	7.79, 6.67	160.47, 139.91
5’	CH	112.06	6.67, d (7.6)	7.36	112.30, 106.71
6’	C	160.47			
7’	C	106.71			
4-OCH_3	CH_3	55.35	3.80, s		160.75

[a]Chemical shifts in ppm.

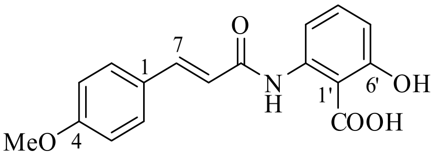

Figure S19. ^{1}H-NMR of compound 3m in $(CD_3)_2SO$

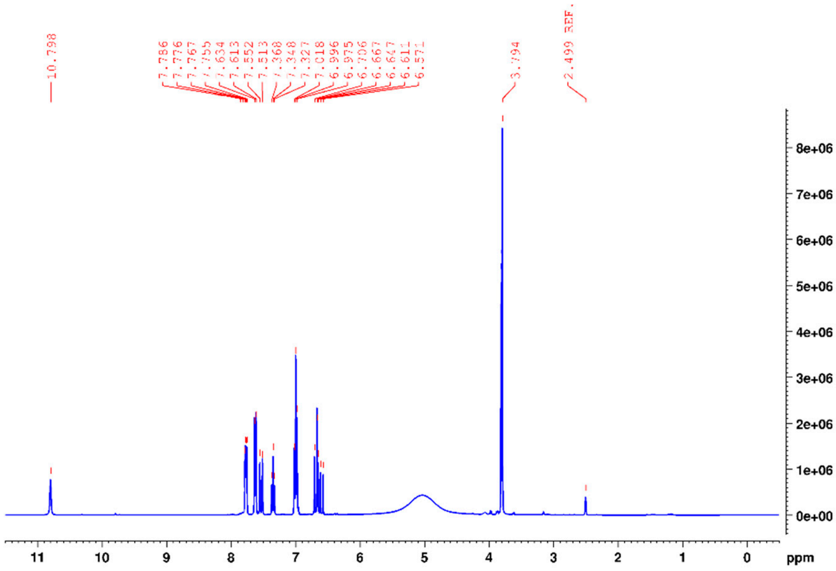

Figure S20. ^{13}C-NMR of compound 3m in $(CD_3)_2SO$

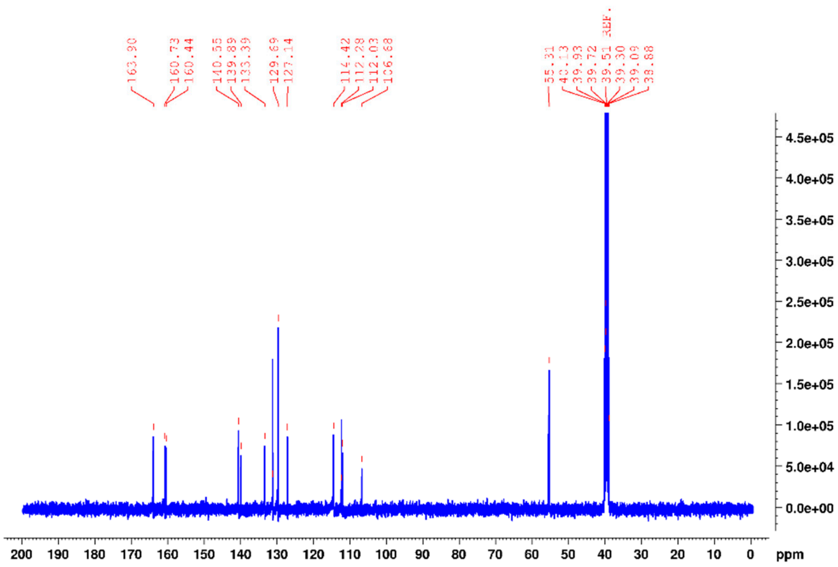

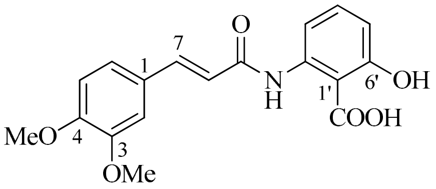

3n: (*E*)-2-(3-(3,4-Dimethoxyphenyl)acrylamido)-6-hydroxybenzoic acid. Beige powder; mp 225-227 °C. ^{1}H- and ^{13}C-NMR: see Table S11. MS-TOF (negative ions): $[M]^-$ calculated for $C_{18}H_{17}NO_6$: *m/z* 343.1056; found 342.3308 $[M - H]^-$ (44%).

Table S11. ^{1}H, ^{13}C and 2D-NMR data of 3n in $(CD_3)_2SO$.

Position	Residue	$^{13}C^a$	$^{1}H^a$, multiplicity (J in Hz)	^{1}H-^{1}H COSY	^{1}H-^{13}C HMBC
1	C	127.54			
2	CH	110.31	7.31, d (2.0)	7.20	152.27, 140.02
3	C	149.04			122.12
4	C	150.27			
5	CH	111.71	6.99, d (8.4)	7.20	149.04, 127.54
6	CH	122.12	7.20, dd (8.4; 2.0)	7.31, 6.99	152.27, 140.02, 110.31
7	CH	140.02	7.51, d (15.8)	6.61	163.66, 122.12, 121.30, 110.31
8	CH	121.30	6.61, d (15.8)	7.51	163.66, 127.54
9	C	163.66			
1’	C	106.29			
2’	C	141.88			
3’	CH	108.35	8.04, dd (8.7; 1.2)	7.15	110.98, 106.29
4’	CH	131.44	7.15, t (8.2)	8.04, 6.44	163.30, 141.88
5’	CH	110.98	6.44, dd (8.2; 1.2)	7.15	108.35, 106.29
6’	C	163.30			
7’	C	173.76			
3-OCH_3	CH_3	55.65	3.84, s		149.04
4-OCH_3	CH_3	55.59	3.80, s		152.27

[a]Chemical shifts in ppm.

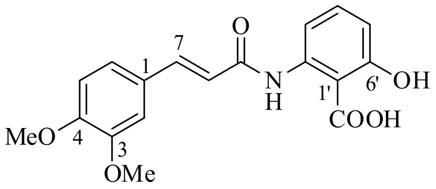

Figure S21. ^{1}H-NMR of compound 3n in $(CD_3)_2SO$

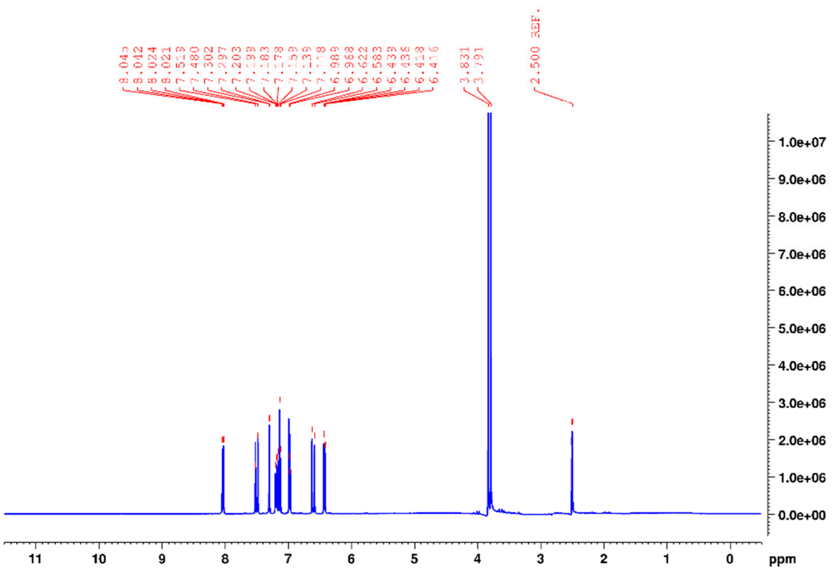

Figure S22. ^{13}C-NMR of compound 3n in $(CD_3)_2SO$

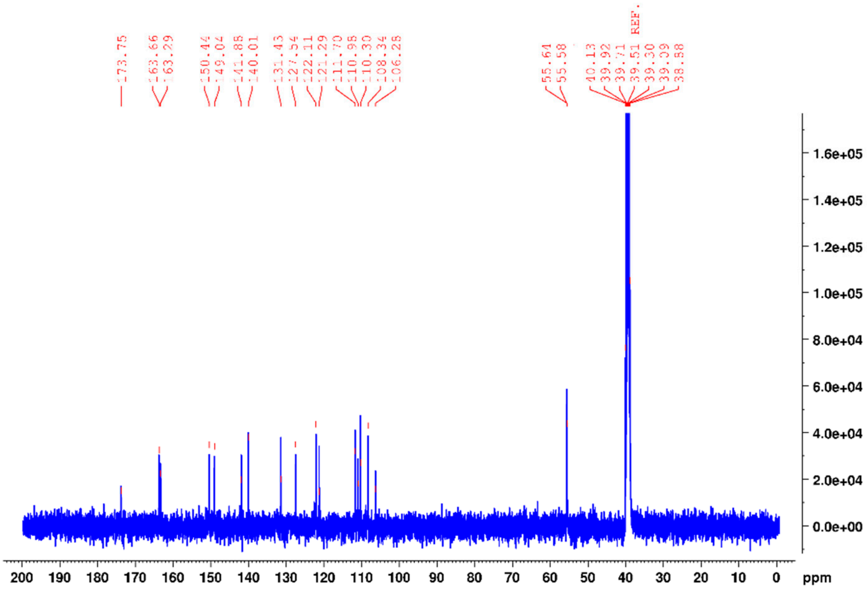

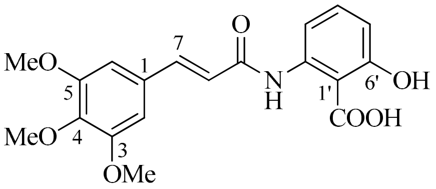

3o: (E)-2-Hydroxy-6-(3-(3,4,5-trimethoxyphenyl)acrylamido)benzoic acid. Beige powder; mp 226-228 °C. ^{1}H- and ^{13}C-NMR: see Table S12. MS-TOF (negative ions): $[M]^-$ calculated for $C_{19}H_{19}NO_7$: *m/z* 373.1162; found 372.3569 $[M - H]^-$ (74%).

Table S12. ^{1}H, ^{13}C and 2D-NMR data of 3o in $(CD_3)_2SO$.

Position	Residue	^{13}C[a]	^{1}H[a], multiplicity (J in Hz)	^{1}H-^{1}H COSY	^{1}H-^{13}C HMBC
1	C	130.26			
2/6	CH	105.52	7.03, s		140.57, 139.02, 105.52
3/5	C	153.13			
4	C	139.02			
7	CH	140.57	7.53, d (16.1)	6.77	163.63, 122.37, 105.52
8	CH	122.37	6.77, d (16.1)	7.53	163.63, 140.57, 130.26
9	C	163.63			
1’	C	106.61			
2’	C	140.74			
3’	CH	110.42	7.91, d (8.1)	7.26	111.62, 106.61
4’	CH	132.38	7.26, t (8.3)	7.91, 6.56	161.77, 140.74
5’	CH	111.62	6.56, dd (8.3; 1.0)	7.26	110.42, 106.61
6’	C	161.77			
7’	C	172.15			
3,5-OCH_3	CH_3	56.02	3.85, s		153.13
4-OCH_3	CH_3	60.12	3.71, s		139.02

[a]Chemical shifts in ppm.

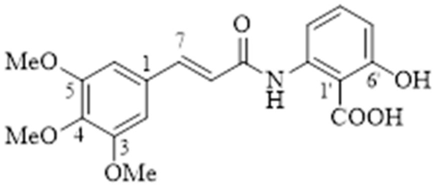

Figure S23. ^{1}H-NMR of compound 3o in $(CD_3)_2SO$

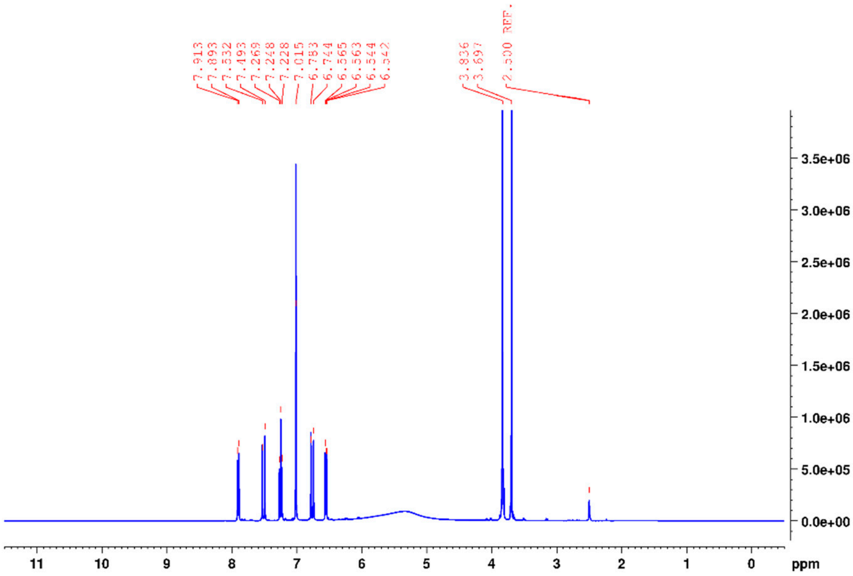

Figure S24. ^{13}C-NMR of compound 3o in $(CD_3)_2SO$

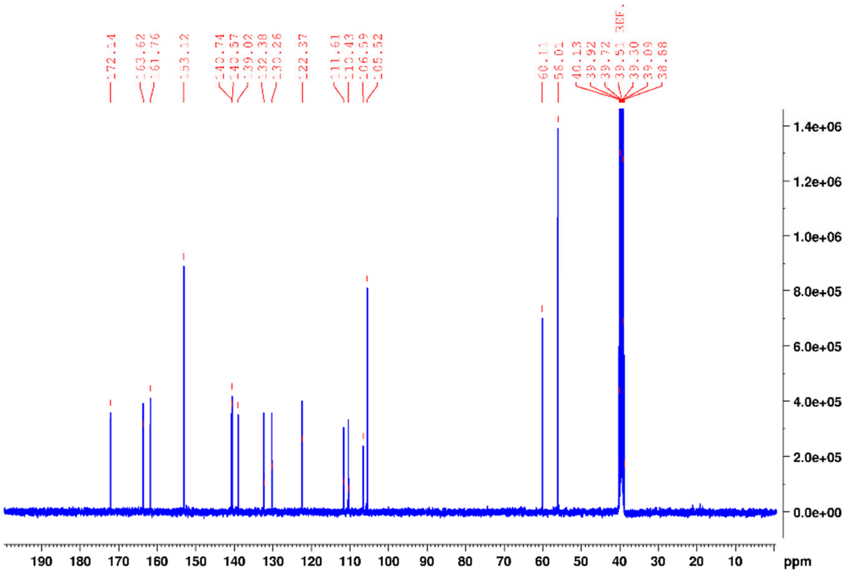

Table S13. Conversion of tested concentrations from µg/mL to µM for compounds 3a-o.

Compound	Molecular weight (g/mol)	50 µg/mL (µM)	100 µg/mL (µM)	150 µg/mL (µM)	200 µg/mL (µM)
3a	297.31	168.17	336.35	504.52	672.70
3b (Tranilast)	327.33	152.75	305.50	458.25	611.00
3c	357.36	139.91	279.83	419.74	559.66
3d	313.31	159.59	319.17	478.76	638.35
3e	343.33	145.63	291.26	436.90	582.53
3f	373.36	133.92	267.84	401.76	535.68
3g	313.31	159.59	319.17	478.76	638.35
3h	343.33	145.63	291.26	436.90	582.53
3i	373.36	133.92	267.84	401.76	535.68
3j	313.36	159.56	319.12	478.68	638.24
3k	343.33	145.63	291.26	436.90	582.53
3l	373.36	133.92	267.84	401.76	535.68
3m	313.31	159.59	319.17	478.76	638.35
3n	343.33	145.63	291.26	436.90	582.53
3o	373.36	133.92	267.84	401.76	535.68

References

1. Zarrelli, A.; Longobardo, L. Eco-friendly synthesis of 2-styryl-benzo[d][1,3]oxazin-4-ones from N-cinnamoyl-anthranilic acids. *Molecules* **2026**, 31, 709. https://doi.org/10.3390/molecules31040709

2. Zarrelli, A.; Longobardo, L. Improved chemical synthesis of avenanthramides family and its analogs by mixed anhydride method. *Eur. J. Org. Chem*. **2025**, 28, e202500169. https://doi.org/10.1002/ejoc.202500169
